# Nursing Work Environments and Patient Safety in Older Adults: A Scoping Review of Their Intricate Relationship, Definitions and Theoretical Frameworks

**DOI:** 10.1111/jan.70683

**Published:** 2026-07-29

**Authors:** Trine Vintersborg, Pelle Korsbæk Sørensen, Lene Halling Hastrup, Lise Marie Thorsboe, Bibi Hølge‐Hazelton, Lene Lauge Berring

**Affiliations:** ^1^ Psychiatric Research Unit Central and West Zealand Hospital Slagelse Denmark; ^2^ Department of Regional Health Research University of Southern Denmark Odense Denmark; ^3^ Centre for Health University College Absalon Roskilde Denmark; ^4^ Danish Center for Health Economics (DaCHE) University of Southern Denmark Odense Denmark; ^5^ Mental Health Services South, Psychiatry in Region Zealand Vordingborg Denmark; ^6^ Research Support Unit Zealand University Hospital Køge Denmark; ^7^ Research Support Unit Zealand University Hospital Roskilde Denmark

## Abstract

**Aim(s):**

To identify and map the relationship between nursing work environments and patient safety for older adults ≥ 65.

**Design:**

Scoping review and consultation exercise.

**Methods:**

Systematic searches of five databases were conducted in 2022, 2024 and 2026. Two reviewers independently screened titles/abstracts and subsequently by full‐text review. Data was extracted using an extraction form with knowledge users involved throughout.

**Results:**

Eighty‐four studies were included. The data were presented with three main categories: (1) study characteristics; (2) core constructs; (3) the relationship between nursing work environments and patient safety, including theoretical frameworks and knowledge users' insights.

**Conclusion:**

The relationship between nursing work environments and patient safety for older adults is intricate. Work environment factors, such as staffing, skill mix and education, influence patient safety, which is defined through outcomes like mortality, readmissions and healthcare‐associated complications.

**Implications:**

Increased awareness of how the work environment influences patient safety can support prioritisation of nursing practice, staffing, skill mix and increase level of education to reduce errors.

**Impact:**

The findings provide an overview of the ways in which work environment factors shape safety for older adults and highlight gaps in measurement and conceptual clarity. The results have implications for healthcare policy, organisational decision‐making and clinical practice. The review was conducted in accordance with the EQUATOR guidelines for PRISMA‐ScR.

**Contributions to the Wider Global Clinical Community:**

- Emphasises the role of supportive work environments in enhancing care quality globally.
- Provides insight for policy and improvements to benefit patient safety.
- Clarifies the scope of research on nursing work environments and patient safety in older adults.

**Patient or Public Contribution:**

Patients and members of the public were involved in designing the study, collecting data and interpreting the findings. Their contributions included reviewing concepts, co‐creating research questions, providing feedback on methodology, inclusion/exclusion, findings and practice implications.

## 1 Introduction

The shortage of nurses worldwide is estimated to amount to around 6 million (World Health Organization 2020), and the recruitment, the retention, and the work environment of nurses are essential in addressing this problem (Williamson et al. 2022). Creating positive work environments is central to both staff sustainability and the broader goals of healthcare systems. This is reflected in the emphasis of the United Nations on the access to quality essential healthcare services and the provision of safe care (United Nations Department of Economic and Social Affairs 2024). Researchers (e.g., Aiken et al. 2018; Chaboyer et al. 2021; Cristina Gasparino et al. 2021) have investigated aspects of the work environment that influence patient safety, but despite its recognised importance, the relationship remains underexplored in the context of older adults (Wiisak et al. 2025), who constitute the largest group of citizens requiring medical treatment and care within the healthcare system (Lønhaug‐Næss et al. 2023). Due to this knowledge gap, there is a need to examine the breadth by identifying, mapping and synthesising the evidence, and a scoping review is suitable for this purpose (Peters et al. 2020).

## 2 Background

Nursing work environments (NWEs) are complex compositions of physical, mental and social health and well‐being and work culture (Organisation and Burton 2010). Recent work by Maassen et al. (2021) identified 36 elements of a positive hospital work environment, including supportive leadership, opportunities for learning, interprofessional collaboration, adequate staffing levels, psychological safety and a culture that values nurses' professional judgement (Maassen et al. 2021). Research within the field further highlights staff members' mental health and well‐being as central components of a healthy workplace (Sermeus et al. 2022; Turnbach et al. 2024).

Several work‐related and organisational factors (Jørgensen et al. 2021; Organisation and Burton 2010), such as nurse‐to‐patient ratios, limited influence on working conditions, heavy workloads and inadequate support, have been shown to negatively affect nurses' mental health (Dall'Ora, Maruotti, and Griffiths 2020; Groves et al. 2023; Shin et al. 2018). This can reduce self‐efficacy and may lead to burnout and low job satisfaction (Dall'Ora, Ball, et al. 2020; Shin et al. 2018), which is a point of concern for the quality of care in both somatic care, originally established in seminal work by Aiken, Sloane, et al. (2011) and mental health care (Hylén et al. 2018; McTiernan and McDonald 2015). Ultimately, this can lead to safety incidents (Stalpers et al. 2015; Steel et al. 2021; Twigg et al. 2015) such as medication errors (Dall'Ora, Ball, et al. 2020; Garcia et al. 2019). This reflects a wider concern about the consequences of stressful working conditions, ranging from diminished well‐being to more severe outcomes, as highlighted by Groves et al. (2023), who identified an increased risk of severe outcomes, including suicide, among nurses in relation to work environment and interpersonal factors.

Patient safety, defined as the reduction of unnecessary harm associated with healthcare to an acceptable minimum (World Health Organization 2021), has gained universal attention following the Institute of Medicine's 1999 report, *To Err is Human* (Institute of Medicine (US) Committee on Quality of Health Care in America 2000). The report emphasised the importance of patient safety and proposed ways of improving it by understanding it as a large, complex problem, which requires multifaceted responses. Furthermore, the report highlighted patient safety as the first critical step to improving quality of care (Institute of Medicine (US) Committee on Quality of Health Care in America 2000). Since then, research has focused on improving patient safety through processes that continuously seek to reduce harm (Bates and Singh 2018; Stelfox 2006).

Contemporary patient safety science increasingly conceptualises safety not merely as the absence of adverse events (Safety‐I), but as the presence of organisational and team capacities that enable everyday work to go well (Safety‐II). From this perspective, safety emerges through professionals' ability to anticipate, adapt and respond to varying conditions in complex environments rather than solely through the prevention of errors (Hollnagel 2018; Hollnagel et al. 2015). Related frameworks emphasise adaptability, psychological safety, learning culture and strong professional relationships as enablers of safe care (Dekker 2016; Woods 2006). Despite progress, patient safety remains a global challenge: one in 10 patients in high‐income countries are exposed to unintended harm, and 13% of total hospital costs in OECD countries are attributed to unintended events, such as infections, medication errors, falls, pressure ulcers and restraint use (Slawomirski et al. 2017; Slawomirski and Klazinga 2022; Tchijevitch et al. 2023; Thibaut et al. 2019).

The relationship between NWEs and patient safety has frequently been described using the Structure‐Process‐Outcome Model (Labrague et al. 2022). Within this model, unfavourable work environments characterised by inadequate resources or deficient leadership directly compromise care processes, including interprofessional collaboration, and lead to poorer outcomes such as adverse patient outcomes and reduced nurse job satisfaction (Labrague et al. 2022). This fuels a negative spiral: low job satisfaction leads to increased turnover intention (Wu et al. 2020), which exacerbates challenges such as insufficient staffing and resource adequacy (Mihdawi et al. 2020). In addition, when leadership is poor, managers are frequently identified as the culprits of the workplace, which reinforces the negative organisational structure that is linked to patient safety (Tlhako et al. 2025). If such negative conditions persist, they risk causing irreversible harm to patient care and nurses' health (Mihdawi et al. 2020).

Over the last 20 years, somatic hospital settings have seen progress (Aiken et al. 2002; Kane et al. 2007) in linking work environment factors to patient safety; however, this review extends beyond somatic care to include evidence from mental health nursing, where psychosocial work environment factors are particularly salient. Adverse events in mental health care can be influenced by structural and cultural factors, staff‐patient interactions and communication. This leads to different patterns of patient outcomes than in somatic care (Bowers et al. 2011; Papadopoulos et al. 2012). Mental health care presents complexities, including behaviours such as self‐harm, aggression and the use of coercive interventions (Thibaut et al. 2019), that require further investigation. Research by Bowers et al. (2009) and Bowers and Crowder (2012) has revealed a multifaceted relationship between staffing levels and adverse events in mental health settings. They found that although higher staffing levels were associated with an increased number of incidents of conflict and aggression, they also corresponded with reduction in self‐harm, medication refusal and the need for pro re nata medication (Bowers and Crowder 2012). These findings align with the study by Fukasawa et al. (2018), which reported more frequent use of seclusion and restraint use in wards with higher nurse staffing levels. In contrast, one study found that the patient‐staff ratio and acceptable work environment positively affected restraint use (Bak et al. 2015). Taken together, these studies illustrate the complexity of staffing dynamics in mental health settings.

Despite these insights, mental health care remains comparatively under‐researched (Thibaut et al. 2019). A systematic review by Moyo et al. (2020) found no eligible studies examining the associations between mental health nurse‐to‐registered nurse (RN) ratios and patient outcomes such as readmissions or referrals to crisis team. In the absence of empirical findings on staffing ratios, research in the Nordic has investigated the potential of milieu therapy and patient‐centred care to improve outcomes, such as reducing restraint use (Bak et al. 2012).

More than five recent reviews attest to the ongoing interest in understanding the relationship between NWEs and patient outcomes. A systematic review by Almutairi and Said found that poor staffing, long hours and weak leadership were associated with missed care and safety risks (Almutairi 2025). Lake et al. (2019) conducted a meta‐analysis which showed that better NWEs reduced burnout, mortality and adverse events and improved satisfaction. Lee and Scott (2018) reported that positive work environments were associated with lower mortality, fewer falls and reduced failure to rescue (FTR), although the results were mixed for medication errors and infections. Li et al. (2024) found that nurse burnout was associated with more errors, infections, falls and missed care and with lower satisfaction. Pryor et al. (2024) highlighted strong links between nurse well‐being, staffing and adverse outcomes such as errors, infections, falls and pressure ulcers. Nascimento and Jesus (2020) concluded that favourable work environments reduce mortality, FTR, errors, infections and pressure ulcers. Further, two systematic reviews have highlighted the importance of nursing‐sensitive indicators (NSIs) for understanding the link between nursing care and patient outcomes, which underlines the need for better staffing and supportive environments to enhance patient safety (Ngune et al. 2023; Oner et al. 2021). Collectively, these syntheses underscore that favourable NWEs are linked to reduced adverse patient events; however, one challenge identified is inconsistency in methodologies, theoretical frameworks and measurement approaches. To address this complexity, NSIs, defined as changes in a patient's health status directly influenced by nursing care (Afaneh et al. 2021), are important for understanding how NWEs can affect patient safety (Afaneh et al. 2021; Mainz et al. 2023).

Safety risks are particularly pronounced in the elderly. This population is growing worldwide and is more susceptible to infections, risks of adverse drug events and microbial causes (Yoshikawa 2000). Their symptoms can present atypically, which may delay diagnosis and treatment (Yoshikawa 2000). Such vulnerabilities place greater demands on nurses, whose work environment plays a significant role in ensuring patient safety. An unsatisfactory work environment contributes to nurse burnout, job dissatisfaction and impaired mental health, all of which can decrease care quality and increase elderly patients' risk of central line‐associated bloodstream infections (CLABSI), ventilator‐associated pneumonia, mortality, decubitus ulcers and catheter‐associated urinary tract infections (CAUTI) (Stone et al. 2007). With multimorbidity affecting approximately 37% of adults worldwide and more than 50% of those aged 60 years and over (Chowdhury et al. 2023), the need to examine age‐specific patient safety outcomes and the role of NWEs is increasingly important.

To our knowledge, this scoping review is the first to provide an overview of studies published about NWEs, patient safety and their relationship across healthcare settings. This knowledge may support nurses and policymakers in preventing adverse events caused by poor working conditions.

## 3 The Review

### 3.1 Aim

The objective was to identify and map empirical studies on the relationship between nursing work environments and patient safety for patients aged 65 years and over in order to provide an understanding of how nursing work environments may impact outcomes in this population.

### 3.2 Review Question

What is reported in empirical studies on the relationship between nursing work environments and patient safety for patients aged 65 years and over across somatic hospitals and secondary psychiatric hospitals?

The review question was formulated using the PCC framework (Population, Concept and Context) (Peters et al. 2020) (Table 1) and specifically focuses on:

- Study characteristics.
- Core constructs of work environment and patient safety, including definitions, measures, key elements, dimensions, measurement tools, outcomes and the implications of multimorbidity (the coexistence of physical and mental health conditions).
- The relationship between NWEs and patient safety, comprising nursing‐sensitive indicators and the theoretical frameworks that describe the relationship.

**TABLE 1:** Eligibility criteria.

Population, concept, context	
Population	Patients aged 65 and over, either as a distinct group or within a broader age range Nurses with the minimum level of education required for RNs
Concept	Nursing work environments Patient safety Nursing sensitive indicators
Context	Hospital‐based settings across all somatic and psychiatric specialities caring for adults aged 65 years and over. This includes all units (e.g., medical, surgical, geriatric, emergency, psychiatric units) with interdisciplinary workflows and dynamic patient trajectories

Inclusion criteria	Exclusion criteria
Objective measures of patient safety or nursing‐sensitive indicators Empirical peer‐reviewed qualitative, quantitative or mixed‐methods studies Published from 2010 Language: English, Danish, Swedish, or Norwegian Geography: Europe, Canada, the United Kingdom, the United States of America and Australia	Studies developing and testing measurement scales Studies examining quality of care, patient satisfaction, perceived patient safety or safety climate experienced by nurses Individuals in nurse leadership, managerial roles, nurse assistants and other healthcare professionals Nursing homes, military hospitals and general practices (due to different organisational structures and clinical characteristics) Conference abstracts, dissertations, opinion papers, reports, guidelines, reviews and policy papers Studies that do not include a patient population aged 65 or older Studies related to COVID‐19 or natural disasters

### 3.3 Methods

The review was conducted following the JBI methodology for scoping reviews, as outlined in the JBI Manual for Evidence Synthesis (Peters et al. 2024), including Pollock et al.'s (2023) recommendations for the extraction, analysis and presentation of results, and is reported according to the Preferred Reporting Items for Systematic reviews and Meta‐Analyses extension for Scoping Reviews (Tricco et al. 2018). The protocol is registered in the Open Science Framework: https://doi.org/10.17605/OSF.IO/247UC and in the a priori protocol (Thorsboe et al. 2023).

The original objective was to provide an overview of factors influencing NWEs and patient safety across multiple healthcare contexts. This review narrows its focus to patients aged 65 and over with the objective of identifying potential NSIs to refine our understanding of how NWEs influence patient safety, including both somatic and mental health care settings.

#### 3.3.1 Knowledge User Engagement

A knowledge user group (Pollock et al. 2022) was established consisting of patients, relatives, RNs, nurse managers and work environment representatives from four regional hospitals in Denmark. Through workshops, the group helped refine the review's focus on work environment and patient safety issues. The group members co‐developed research questions and contributed to defining inclusion criteria, thereby ensuring alignment with clinical practice. They provided feedback on the protocol, data extraction and preliminary findings.

See Supporting Information for an overview of the specific activities involving knowledge users.

#### 3.3.2 Eligibility Criteria

To ensure transparency, clear inclusion and exclusion criteria have been established (Table 1).

#### 3.3.3 Search Strategy

A search of MEDLINE (PubMed) and CINAHL (EBSCO) was performed to identify relevant articles. Based on this, a comprehensive search strategy was developed using words in titles and abstracts, keywords and index terms from the identified studies, with additional input from knowledge users to refine relevant search terms. Subsequently, the search strategy was tailored for each database, including MEDLINE (PubMed), Web of Science (Clarivate), Embase (OVID), APA PsycINFO (OVID) and CINAHL (EBSCO). Throughout the process, a clinical librarian was consulted, who guided the selection of databases, keywords and the development of the search strategy.

The search did not include restrictions on the publication date; however, due to the comprehensive nature of the results to be assessed, the research team decided to exclude studies published before 2010 to include the most recent literature and ensure contemporary NWEs and patient safety practices. Developments in healthcare organisations have occurred in the past decade, making earlier studies less comparable to current conditions.

#### 3.3.4 Study Selection

The studies retrieved were exported to Zotero (version 6.0) for reference management. From the original search in 2022, a total of 8153 references were retrieved and imported into the Covidence Systematic Review software. Updated searches identified 1480 references in 2024 and 1899 in 2026. A pilot test was conducted by three reviewers (T.V., L.M.T., L.L.B.) to screen titles and abstracts and ensure agreement. Titles, abstracts and subsequent full‐text screenings were performed independently by two researchers (T.V., L.M.T.). The updated searches were screened independently by three researchers (T.V., L.L.B., P.K.S.). Conflicts were resolved through discussion until consensus was reached. For an overview of references identified, included and excluded, see Figure 1.

**FIGURE 1:**
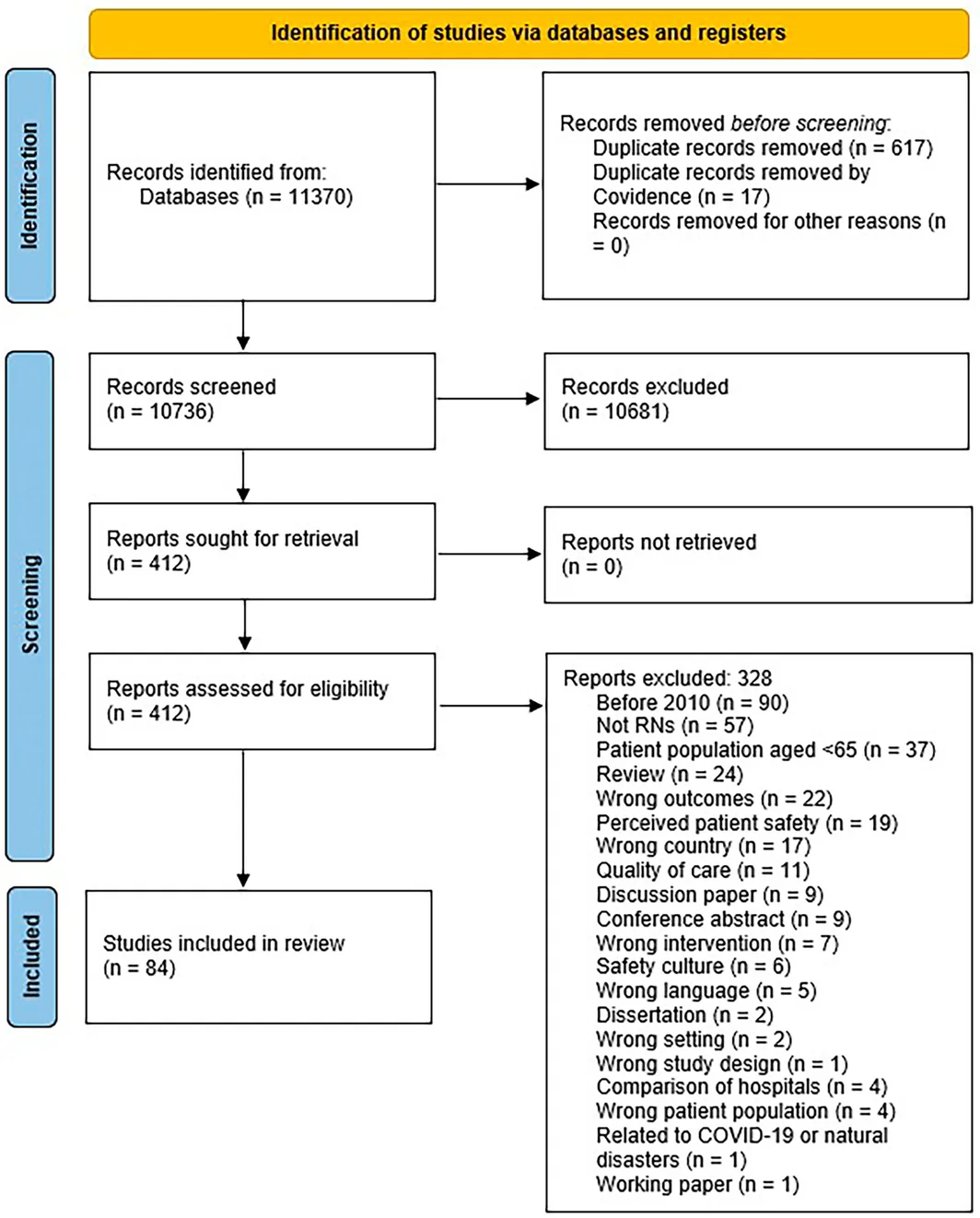
PRISMA flowchart.

#### 3.3.5 Data Extraction

The data was extracted in the Covidence Systematic Review software by two reviewers (T.V., L.M.T.) using a draft data extraction form developed by the reviewers and pilot tested on 25 articles (T.V., L.M.T.). Disagreements were solved through discussion or by involving the research team. Following the principles by Pollock et al. 2023, an extraction guidance form was developed to accompany the final extraction form, which included information about author(s), title of source, publication, year, date data were collected and type of evidence source. Lastly, the data extraction form was designed, guided by the review question and the PCC framework. An Excel spreadsheet was created to chart the relevant data. This included author(s), year, country, aim, study design, population (patients and nurses), health diagnosis, context (hospital setting and unit), concept (patient safety outcomes and work environment), impact of NWEs on patient safety and multimorbidity.

#### 3.3.6 Quality Appraisal

Given that scoping reviews provide an overview of evidence regardless of study quality, the included literature has not undergone critical appraisal in accordance with JBI (Peters et al. 2015).

## 4 Findings

A total of 11,370 studies were identified. After 634 duplicates had been removed (17 identified manually and 617 identified by Covidence), 10,736 articles remained for screening. Of these, 10,681 were deemed irrelevant and excluded. The remaining 412 articles underwent full‐text screening, which resulted in the exclusion of 328 articles. A total of 84 studies met the inclusion criteria.

### 4.1 Study Characteristics

*Study type*: Only quantitative studies were included (*n* = 84).

*Country of origin*: The research had been conducted in 15 different countries; most of the studies had been conducted in the United States of America (*n* = 52), followed by the United Kingdom (*n* = 7), Australia (*n* = 5), Canada (*n* = 3), Finland (*n* = 3), Belgium (*n* = 3) and Spain (*n* = 2). One study had been conducted in each of the following countries: Germany, Poland, Sweden, the Netherlands, Greece, Italy and Switzerland (*n* = 1). A broader European perspective was provided by RN4CAST (*n* = 2), which covered multiple countries.

*Setting*: The units studied were surgical (*n* = 60), medical (*n* = 36), intensive care/critical care (*n* = 28), medical‐surgical (*n* = 25), step‐down (*n* = 12), rehabilitation (*n* = 6), general (*n* = 8), acute care (*n* = 6), orthopaedic (*n* = 2), emergency medicine (*n* = 2), paediatric (*n* = 1), gynaecology (*n* = 1), maternity (*n* = 1), neurology (*n* = 1), oncology (*n* = 1), lung (*n* = 1), otology (*n* = 1), telemetry (*n* = 1) and mental health care (*n* = 1).

*Patient age*: Thirteen studies focused solely on elderly people (65 years and older); 62 included both adults and elderly individuals (age 18–65 and older); and nine encompassed three age groups (children aged 0–17, adults and elderly).

### 4.2 Core Constructs in the Work Environment of Nurses and Patient Safety

Core constructs refer to the foundational elements that define a concept; in this case, they are NWEs and patient safety. This section describes how NWEs and patient safety are defined and measured, and it outlines key elements, dimensions and measurement tools related to the work environment, how patient safety is described and understood, and also outcomes. Multimorbidity will be explored to examine its influence on the complexity of care.

#### 4.2.1 The Nursing Work Environment

The work environment for nurses was implicitly defined in 76% of the studies (*n* = 64), whereas 24% (*n* = 20) provided an explicit definition. An implicit definition is inferred from the variables, measures or contextual descriptions that the authors used to operationalise it, whereas an explicit definition is stated directly. Explicit definitions are listed in Table 2.

**TABLE 2:** Explicit definitions of NWEs.

Author(s)	Definition of work environment
Olds et al. (2017)	The set of organisational characteristics of a work setting that facilitate or constrain professional nursing practice
Catania et al. (2024)	The presence of some organisational characteristics of the work environment in the healthcare context that facilitate or hinder nursing practice
Ma and Park (2015)	Organisational factors at different structural levels (e.g., *hospital level or unit level*), which can facilitate or constrain professional nursing practice
McHugh et al. (2016)	Greater autonomy, control over practice and resources, and excellent working relationships and communication with physicians
Aiken, Cimiotti, et al. (2011)	Single composite measure of the practice environment formed by averaging four aggregated subscales of the Practice Environment Scale of the Nursing Work Index (PES‐NWI), excluding staffing/resource adequacy
Kelly et al. (2014)	Score of four Practice Environment Scale of the Nursing Work Index (PES‐NWI) subscales (*staffing and resource adequacy subscales excluded*)
Dubois et al. (2013)	Five subdimensions of The Nursing Work Index: nurse participation in hospital affairs, nursing foundation for quality, nurse manager leadership and support, resource adequacy and nurse‐physician relations
Lasater and Mchugh (2016)	Practice Environment Scale of the Nursing Work Index (PES‐NWI)
Kumar et al. (2022)	Practice Environment Scale of the Nursing Work Index (PES‐NWI) (*staffing and resource adequacy subscales excluded*)
Rao et al. (2017)	Nurse autonomy as part of professional work environments
Choi et al. (2013)	Structural characteristics, such as Magnet status, teaching status, bed size, which are related to RN workgroup job satisfaction
Schlak et al. (2021)	Improved nurse autonomy, better staffing and supportive management
Bae et al. (2010)	Work complexity (e.g., *frequent physician order changes*, *admissions*, *transfers*, *discharges*) and the availability of support services
Daus et al. (2024)	Practice Environment Scale of the Nursing Work Index (PES‐NWI) focusing on the subscales: hospital affairs, nursing foundations, MNALS, N‐P relations and SRA
Ball et al. (2018)	The ‘Practice Environment Scale’ (PES‐28) is measured using 28 items from the Nursing Work Index, where four items related to perceptions of nurse staffing were excluded
Frith et al. (2010)	Workloads are unsafe when the nursing units are understaffed and nurses are caring for frail, elderly patients with high technology demands
Park et al. (2012)	Good environmental structure to provide safe and high‐quality care in the health care sector
Xue et al. (2012)	National Database of Nursing Quality Indicators (NDNQI)‐adapted Index of Work Satisfaction, and categorised into three levels: ‘better, mixed and poorer work environment’
Bettencourt et al. (2020)	Clinical work environment using Practice Environment Scale of the Nursing Work Index (PES‐NWI) scale scores and its subscales
Tack et al. (2026)	Nursing workload measured using the Nursing Activities Score (NAS) at ICU discharge

NWEs were described as follows: staffing levels were a focus in 61% of the studies (*n* = 51) (e.g., Ambrosi et al. 2017; Dall'Ora, Maruotti, and Griffiths 2020; Dietermann et al. 2021; Duffield et al. 2011; Moronski et al. 2025; Muir et al. 2026; Rochefort et al. 2022) as was skill mix in 38% (*n* = 25) (e.g., Ambrosi et al. 2017; Boltz et al. 2013; Esparza et al. 2012; He et al. 2016; Kutney‐Lee et al. 2017). Both the physical and psychological aspects of the workplace (e.g., Lake et al. 2010) including safety standards, resource availability (Wieczorek‐Wojcik et al. 2020), leadership and peer support (e.g., Bae et al. 2010; Olds et al. 2017; Rao et al. 2017; Salvador et al. 2021; Schlak et al. 2021), were noted in relation to retention.

In 39% of the studies, there was a focus on nursing education (e.g., Aiken, Cimiotti, et al. 2011; L. Aiken et al. 2014; Boltz et al. 2013; Harrison et al. 2019; Iroegbu et al. 2026; Juvé‐Udina et al. 2020) (*n* = 33) and in 4% on certification (Boltz et al. 2013; Kendall‐Gallagher et al. 2011; Lake et al. 2010) (*n* = 3). The studies highlighted higher levels of education, particularly nurses with Bachelor of Science in Nursing (BSN) degrees (Kendall‐Gallagher et al. 2011), expertise (Juvé‐Udina et al. 2020) and experience (Rochefort et al. 2020), in relation to beneficial outcomes for both nurses and patients. In addition, the roles and qualifications of nurses were examined. RNs were reported in 83% of the studies (*n* = 70), and the remaining 17% (*n* = 14) included advanced practice nurses, nurse practitioners or BSN‐educated nurses as part of a broader interdisciplinary group of healthcare professionals.

A range of factors were examined as components of the work environment, such as nurse absenteeism, turnover rates (e.g., Boltz et al. 2013; Moscelli et al. 2024; Park et al. 2014; Scheier et al. 2021) (*n* = 7) and the presence of agency or casual staff (e.g., Bae et al. 2010; Dall'Ora, Maruotti, and Griffiths 2020) (*n* = 7). Seven per cent of the studies (*n* = 6) also emphasised the role of job satisfaction (e.g., Choi et al. 2013; Dzikowicz et al. 2020; Rao et al. 2017) and intent to leave (e.g., Aiken, Cimiotti, et al. 2011) (*n* = 3). The usability of electronic health records focusing on factors influencing workflow efficiency, job satisfaction and care delivery was explored (Kutney‐Lee et al. 2021). Moreover, the flexibility of staff was examined as a component of adaptability and organisational responsiveness (Salvador et al. 2021).

Burnout among nurses (e.g., Aiken, Cimiotti, et al. 2011; Kutney‐Lee et al. 2021; Park et al. 2014; Schlak et al. 2021) was investigated in 6% of the studies (*n* = 5), and one examined how the work environment impacted burnout levels (Schlak et al. 2021). Environmental turbulence (Park et al. 2012) (e.g., frequent admissions, discharges or transfers) can lead to chaotic working conditions, which may strain resources and increase the risk of burnout. A professional work environment with autonomy (McHugh et al. 2016; Rao et al. 2017) (*n* = 2), management support (Olds et al. 2017) and good interdisciplinary collaboration (McHugh et al. 2016) were highlighted as factors in relation to burnout and retention.

In 21% of the studies (*n* = 18), workload (e.g., Aiken, Cimiotti, et al. 2011; L. Aiken et al. 2014; Cottney and Innes 2015; Duffield et al. 2011; Fasoi et al. 2020; Tack et al. 2026) was addressed in the context of nurse well‐being, as was physical work environment metrics, including the quality of equipment and adherence to safety standards. Workload balance (Giuliano et al. 2016) was examined as a component of a healthy work environment with implications for both nurses and patients. High patient turnover (Park et al. 2012), interruptions (Cottney and Innes 2015; Drews et al. 2019) and workload were found to contribute as stressors that impact nurse well‐being and the quality of care.

An overview of the identified dimensions, key elements and corresponding examples is presented in Table 3, which presents an aggregated synthesis of both constraining and enabling NWE features.

**TABLE 3:** Dimensions of the NWE, key elements and examples of studies.

Work environment dimensions	Key elements of the work environment	Examples of studies
Staffing levels and skill mix	Nurse staffing Patient‐to‐Nurse Ratios Nursing Hours Per Patient Day (*NHPPD*) Skill mix	[3, 11, 13, 14, 20, 25, 27, 32, 33, 34, 35, 36, 37, 39, 40, 42, 43, 44, 46, 48, 50, 52, 54, 56, 57, 59, 60, 62, 67, 68, 70, 75, 74]
Workload and work complexity	Patient care needs High patient turnover Environmental turbulence Nurse workload Distractions/interruptions Flexibility	[18, 29, 34, 37, 42, 49, 55, 75]
Interpersonal relationships and teamwork	Managerial support Nurse‐physician relations Co‐worker support	[26, 24]
Organisational factors/administrative factors	Impact of technology Management support Level of education/certification	[14, 53, 57, 60, 70]
Psychosocial factors	Burnout Autonomy Job satisfaction	[73, 65, 61, 51]
Environmental characteristics	Unit size Nurse shortages Temporary staffing Availability of support services Turnover	[76, 58, 55]
Positive NWEs (*enabling features across dimensions*)	Adequate RN staffing Skill mix Manageable workload Supportive leadership Collegial collaboration Nurse autonomy Psychological safety Continuity	[8, 12, 21, 24, 25, 47, 51, 52, 64, 65, 69, 71]

*Note:* The numbers in square brackets represent reference numbers and are listed in the Supporting Information.

Eight different assessment tools were identified, by which NWEs were evaluated or measured. These included the Practice Environment Scale of the Nursing Work Index ‘PES‐NWI’ (e.g., Bettencourt et al. 2020; Ma and Park 2015; Wiltse Nicely et al. 2013) and the Nursing Work Index‐Revised ‘NWI‐R’ (e.g., Duffield et al. 2011). These scales focus on dimensions such as nurse participation in decision‐making, staffing adequacy and relationships with physicians. Other scales included the Environmental Complexity Scale ‘ECS’ (Duffield et al. 2011), the RAFAELA System (Fagerström et al. 2018), the PAONCIL Method (Fagerström et al. 2018), the OULU Patient Classification (Fagerström et al. 2018), the NDNQI adapted Index of Work Satisfaction ‘IWS’ (Xue et al. 2012) and Inventario del Nivel de Cuidados mediante Indicadores de Clasificación de Resultados de Enfermería ‘INICIARE’ (Porcel‐Gálvez et al. 2020). These assess patient acuity, care intensity, organisational complexity and working conditions.

#### 4.2.2 Patient Safety

Implicit definitions of patient safety were provided in 73% (*n* = 61) of the studies, whereas 27% (*n* = 23) offered an explicit definition. Patient safety was operationalised through the outcome measures, such as mortality or falls, which indicates that measurement functioned as the primary articulation of how patient safety was defined/understood. Thus, the boundaries between definitions and outcome measures were often indistinct.

Patient safety outcomes were assessed using various measures. Mortality (e.g., Falk 2022; Fogg et al. 2021; Haegdorens et al. 2019; Kendall‐Gallagher et al. 2011; Lasater et al. 2025; Trinkof et al. 2011), which was present in 57% of the studies (*n* = 48), was the most frequently reported outcome, followed by cases of hospital‐acquired pressure ulcer (HAPU)/decubitus ulcer in 27% (e.g., Dietermann et al. 2021; He et al. 2016; Salvador et al. 2021) (*n* = 23), length of stay (LOS) in 25% (*n* = 19) (e.g., Falk 2022; Lasater, McHugh, et al. 2021b; Lasater, Sloane, et al. 2021; Roberts et al. 2022) and FTR in 17% (*n* = 14) (e.g., Kendall‐Gallagher et al. 2011; Rao et al. 2017; Twigg et al. 2016). Occurrences were documented for falls (*n* = 12) (e.g., Boltz et al. 2013; Duffield et al. 2011; Fernández‐Sánchez et al. 2025; He et al. 2016; Lake et al. 2010; Twigg et al. 2016), readmission (*n* = 16) (e.g., Dzikowicz et al. 2020; Giuliano et al. 2016; Lasater, McHugh, et al. 2021b, Lasater, McHugh, et al. 2021a; Lasater and Mchugh 2016; Moronski et al. 2025) and medical errors (*n* = 8) (e.g., Bae et al. 2010; Cottney and Innes 2015; Fernández‐Sánchez et al. 2025). CAUTI/UTI were reported (*n* = 9) (e.g., Dzikowicz et al. 2020; Esparza et al. 2012; Frith et al. 2010; Schreuders et al. 2015; Twigg et al. 2016), as were sepsis (*n* = 7) (e.g., Duffield et al. 2011; Flanagan et al. 2020; Lasater, Sloane, et al. 2021), pneumonia (*n* = 8) (e.g., Dietermann et al. 2021; Duffield et al. 2011; Meredith et al. 2024; Schreuders et al. 2015; Twigg et al. 2012), CLABSI (*n* = 5) (e.g., Dzikowicz et al. 2020; Scheier et al. 2021) and injurious falls (*n* = 5) (e.g., Boltz et al. 2013; Dzikowicz et al. 2020; Twigg et al. 2016). Other outcomes included shock/cardiac arrest (*n* = 4) (e.g., McHugh et al. 2016; D. Twigg et al. 2012) and restraint use (*n* = 4) (e.g., Boltz et al. 2013; Hart and Davis 2011; Staggs et al. 2017). The potential harm that may be caused by restraint use, such as agitation, confusion, strangulation and death and the need for alternative management strategies (Staggs et al. 2017) were addressed. Hospital‐acquired conditions (HACs) were also a focus, particularly LOS, healthcare costs (Li et al. 2016), infection (Scheier et al. 2021) and mortality (Shang et al. 2019), the risk of which is higher for vulnerable populations such as older adults (Ambrosi et al. 2017; Boltz et al. 2013; Choi et al. 2013; Fogg et al. 2021; Kumar et al. 2022).

In total, the review captured 39 distinct patient safety outcomes recorded with various occurrences.

Table 4 provides an aggregated overview of the primary patient outcome categories.

**TABLE 4:** Primary patient outcomes and examples of studies.

Primary patient outcome categories	Patient safety outcomes	Examples of studies
Mortality‐related outcomes	Mortality 30‐day survival Failure to rescue Unplanned extubation Survival following an in‐hospital cardiac arrest	[8, 10, 14, 15, 16, 17, 19, 20, 21, 22, 24, 25, 27, 28, 29, 30, 32, 33, 35, 36, 37, 38, 39, 40, 43, 45, 51, 53, 54, 55, 60, 63, 69, 70, 71, 72, 74, 75]
Length of stay	Length of stay	[8, 17, 24, 35, 37, 40, 41, 42]
Patient injury outcomes	Hospital‐acquired pressure ulcer Fall/injurious falls Restraint use Other hospital‐acquired injuries (e.g., *air embolism*, *blood incompatibilities*)	[9, 11, 13, 18, 19, 24, 25, 26, 28, 31, 32, 35, 36, 41, 42, 44, 58, 70, 73]
Readmission rates	Readmission (*hospital readmission within a specific timeframe*)	[4, 6, 12, 15, 19, 23, 34, 39, 74]
Care process failures and safety incidents	Medical errors Delirium Missed nursing care Protocol nonadherence and delays in care	[9, 26, 28, 33, 41, 44, 66, 70]
Infections	Catheter‐associated urinary tract infection/urinary tract infection Central line‐associated blood stream infection Pneumonia Postoperative sepsis	[2, 25, 36, 26, 35, 42, 43]
Complications and derangements	Physiological/metabolic derangement Postoperative complications (e.g., *deep vein thrombosis/pulmonary embolism* (*DVT/PE*), *hip fractures*) Other complications (e.g., *gastrointestinal bleeding*, *cardiac arrest*)	[9, 24, 29, 33]
Other outcomes	Patient satisfaction Risk of family/caregiver compassion fatigue Neurologic outcomes (*based on* e.g., *CPC scores*) 30‐day complications (*related to overall patient care*) Assaults Departure against medical advice	[50, 53, 70, 73, 84]

*Note:* The numbers in square brackets represent reference numbers and are listed in the Supporting Information.

Table 5 presents an overview of the most frequently reported measures for both the NWE and patient safety. Measures appearing in two or more studies are included.

**TABLE 5:** Overview of the most frequently reported work environment and patient safety measures.

Most frequently reported measures	
	Count (*n*)
*Work environment*	
Staffing level (e.g., *nursing hours per patient day*, *total nursing hours*, *understaffing*)	46
Level of education (e.g., *proportion of nurses with a bachelor's degree*)	33
Staffing ratio (e.g., *patient‐to‐nurse ratio*)	31
Skill mix (e.g., *proportion of registered nurse hours*, *proportion of registered nurses relative to other personnel*)	25
Work environment (*overall scales such as PES‐NWI*, *IWS*, *ECS*, *work environment complexity*, *high‐intensity work environments*)	24
Physical work environment	19
Level of experience (e.g., *years of experience*, *proportion of experienced nurses*, *RN tenure*)	19
Mental work environment	18
Nurse workload (e.g., *patient care needs*, *patient acuity*, *total number of patients in the unit*)	18
Job satisfaction	6
Turnover	7
Burnout	5
Certification (e.g., *speciality nurse certification*)	3
Intention to leave	3
Nursing autonomy	2
Shift work (e.g., *rotating shifts*)	2
*Patient safety*	
Mortality	48
Hospital‐acquired pressure ulcer/decubitus ulcer	22
Length of stay	19
Readmission	16
Failure to rescue	14
Falls	11
Catheter‐associated urinary tract infection/urinary tract infection	9
Pneumonia	8
Sepsis	7
Medical errors	7
Central line‐associated blood stream infections	5
Injurious falls	5
Restraint use	4
Shock/cardiac arrest	4
Postoperative deep vein thrombosis/pulmonary embolism	3
30‐day survival	2

##### 4.2.2.1 Multimorbidity and Its Implications for Patient Safety

The areas of health investigated among patients included physical health in 75% of the studies (*n* = 63), mental health in 4% (*n* = 3) and multi‐diagnosis in 15% (*n* = 13). The remaining studies did not specify. Five studies specifically acknowledged the presence of multimorbidity and evaluated the concurrence of mental and physical health conditions. Ninety‐two per cent of the studies (*n* = 77) focused only on physical health outcomes without exploring the interaction with mental health. Across the studies, multimorbidity was often addressed indirectly (e.g., L. Aiken et al. 2014; Lasater and Mchugh 2016; Staggs et al. 2017; Wieczorek‐Wojcik et al. 2020) or incorporated into statistical models (e.g., Ball et al. 2018; Lasater, McHugh, et al. 2021b, Lasater, McHugh, et al. 2021a; Lasater, Sloane, et al. 2021; Margadant et al. 2020).

The Charlson Comorbidity Index (*n* = 19) (Charlson et al. 1987) or the Elixhauser Comorbidity Score (*n* = 22) (Elixhauser et al. 1998) were utilised in 49% of the studies (*n* = 41). These instruments assess dementia, heart failure, hypertension, diabetes, neurological disorders, psychoses and depression, and therefore capture both physical and mental health conditions. However, only a limited number of studies examined how multimorbidity might modify the relationship between NWEs and patient outcomes.

Mental health conditions, specifically depression and cognitive impairment (CI), were linked to increased vulnerability to adverse events. Such conditions can contribute to confusion, agitation and reduced ability to participate in care, which increases the risk of falls, skin injuries and incomplete monitoring (e.g., vital signs or glucose levels) or hygiene (e.g., skin care); this is often registered as errors of omission (Juvé‐Udina et al. 2020). CI heightens the complexity of care delivery, as patients with CI require more attention, tailored care and closer monitoring, and it is linked to poorer outcomes, including higher in‐hospital mortality (Porcel‐Gálvez et al. 2020). Depression amplifies the negative impact of inadequate nursing staffing, whereas higher staffing levels, supportive work environments and hospitals with more BSN‐educated nurses can mitigate the risks (Kumar et al. 2022).

### 4.3 The Work Environment‐Patient Safety Relationship

This section presents an overview of the theoretical frameworks that were used to underpin the relationship between NWEs and patient safety. Furthermore, it explores how this relationship was reported and offers insights provided by the group of knowledge users.

#### 4.3.1 Theoretical Frameworks of the Nursing Work Environment‐Patient Safety Relationship

Overall, 10 different theoretical frameworks were used. These models integrate organisational factors, care processes, patient outcomes, staffing levels, leadership, adaptability, ergonomics, organisational responsiveness and workforce dynamics. For an overview of theories and frameworks used and how they were used, see Table 6.

**TABLE 6:** Theoretical frameworks.

Theory or framework	How it was used	Count (*n*)	Studies
Donabedian's Structure‐Process‐Outcome Model	How the structure of care affects the processes of care, which affect the outcomes of care.	5	[61, 44, 35, 52, 29]
Patient Care System Model	To examine the effect of nurse variables, workload and environmental complexity on patient, nurse and system outcomes.	2	[9, 36]
Conceptual Framework of Employee Flexibility	To determine whether an organisation has employees with a variety of skills applied in a variety of work contexts.	1	[18]
Granger Causality and Longitudinal Framework	To examine how past values of explanatory variables (lags) can predict the outcome variable.	1	[31]
Human Factors and Ergonomic Systems Theory	To evaluate work systems, processes and outcomes in response to adverse events.	1	[41]
Magnet Hospital Model (*Aiken*, *Sochalski & Lake*)	To link organisational factors to nurse and patient outcomes.	1	[57]
Structural Contingency Theory	How organisational performance is influenced by the complexity of the environment.	1	[62]
The Quality Health Outcomes Model (Mitchell et al. 1998)	System and patient factors mediate the relationship between clinical interventions and patient outcomes.	1	[52]
April 2019 model	To calculate an estimated risk of death for patients based on age, diagnosis, method of admission and comorbidities.	1	[7]
Aiken's theoretical framework	To examine the relationship between nurse and patient outcomes.	1	[5]

*Note:* The numbers in square brackets represent reference numbers and are listed in the Supporting Information.

#### 4.3.2 Connecting Nursing Work Environments and Patient Safety

##### 4.3.2.1 Staff Retention and Care Continuity

High turnover rates of nursing staff are related to poorer patient outcomes, including higher rates of pressure ulcers and mortality (Duffield et al. 2011; Park et al. 2014). Stability in nursing staff encourages safer care environments; however, although flexibility among nurses may improve adaptability, it may also increase certain risks, including pressure ulcers (Dietermann et al. 2021). Increased use of temporary or agency nursing staff was reported to have a negative impact: higher rates of adverse events and patient deaths were observed in units with significant external temporary RN staffing (Bae et al. 2010; Dall'Ora, Maruotti, and Griffiths 2020). Instances of missed nursing care have been reported in connection with higher mortality rates (Fagerström et al. 2018; Wieczorek‐Wojcik et al. 2020) and outcomes such as infections and pressure ulcers (Salvador et al. 2021), and structural factors such as inadequate resources and high patient turnover were contributing factors (Juvé‐Udina et al. 2020; Salvador et al. 2021). Missed nursing interventions can further exacerbate the risks for elderly patients with issues such as confusion (Ambrosi et al. 2017).

##### 4.3.2.2 Nurse Staffing

Low staffing ratios were linked to higher mortality and increased rates of hospital‐acquired infections (HAIs) (Needleman et al. 2020; Shang et al. 2019). The risks are particularly pronounced among patients with multimorbidity—who are, furthermore, facing higher mortality on weekends when nursing coverage is reduced (Ambrosi et al. 2017; Meredith et al. 2024) ‐ and among patients with CI (Fogg et al. 2021). Higher patient‐to‐nurse ratios were linked to increased readmissions among patients with intellectual and developmental disabilities (Moronski et al. 2025), higher mortality among patients with acute kidney injury (Iroegbu et al. 2026), and increased likelihood of patients leaving care against medical advice (Muir et al. 2026). Inadequate staffing is frequently linked to higher rates of falls, medication errors and extended LOS (Lasater, McHugh, et al. 2021b; Lasater, McHugh, et al. 2021a; Lasater, Sloane, et al. 2021; Rochefort et al. 2020). A higher patient‐to‐nurse ratio increases the odds of in‐hospital mortality and readmission, particularly among patients with CI (Fogg et al. 2021) or depression (Kumar et al. 2022), whereas higher NHPPD and increased RN hours are linked to shorter LOS, fewer UTIs and reduced mortality (Esparza et al. 2012; Haegdorens et al. 2019; Wieczorek‐Wojcik et al. 2020). Across settings, higher RN staffing and a richer skill mix are linked to reduced mortality (Bettencourt et al. 2020; Twigg et al. 2016), fewer adverse events (Bae et al. 2010; Dubois et al. 2013; Frith et al. 2010; He et al. 2016; Shang et al. 2019; Staggs et al. 2017; Twigg et al. 2012), lower rates of readmissions (Giuliano et al. 2016; Lasater, McHugh, et al. 2021b; Lasater, McHugh, et al. 2021a; Ma et al. 2015; Meredith et al. 2024) and decreased incidence of HACs and complications (Twigg et al. 2012). Adequate staffing levels and the role of RNs are discussed as critical factors in maintaining safety standards (Dall'Ora, Maruotti, and Griffiths 2020; Frith et al. 2010), and sufficient staffing is described as an essential component to lower risks (Dall'Ora, Maruotti, and Griffiths 2020; Giuliano et al. 2016; Margadant et al. 2020; Wieczorek‐Wojcik et al. 2020). Furthermore, in critical care settings improved staffing is linked to reduced mortality among mechanically ventilated patients (Kelly et al. 2014).

##### 4.3.2.3 Safety Climate

Studies show that a positive work environment interacts with better patient outcomes (Ma et al. 2015; McHugh et al. 2016). Positive work environments with supportive management, job satisfaction, adequate staffing and improvements in the safety climate, are mentioned in connection with lower mortality and fewer complications (Olds et al. 2017). Focusing on the nursing workload that patients generate is important (Margadant et al. 2020), as this is related to higher mortality rates, particularly when the complexity of care may affect the relationship between nursing practices and patient safety (Kumar et al. 2022). High burnout and job dissatisfaction undermine patient safety and care quality (Catania et al. 2024; Schlak et al. 2021) and are compounded by system‐level challenges such as poor health record usability, which is linked to higher surgical patient mortality and 30‐day readmissions (Kutney‐Lee et al. 2021). In critical care contexts, nursing workload at the point of discharge was shown to be a predictor of subsequent patient deterioration and unplanned readmissions (Tack et al. 2026).

##### 4.3.2.4 Education and Magnet Status

Hospitals employing higher proportions of BSN‐educated nurses tend to exhibit better survival rates and improved patient outcomes (Ball et al. 2018; Harrison et al. 2019; Kelly et al. 2014; Kendall‐Gallagher et al. 2011; Kumar et al. 2022; Lake et al. 2010; Rochefort et al. 2020; Salvador et al. 2021). Moreover, hospitals with Magnet recognition demonstrate better work environments, which leads to lower rates of falls and higher patient satisfaction (Lake et al. 2010; Ma and Park 2015; Schlak et al. 2021).

##### 4.3.2.5 Nursing Sensitive Indicators

To clarify how conditions in NWEs translate into patient safety, NSIs offer patient outcomes that are directly impacted by the quality and quantity of nursing care provided (Catania et al. 2024; Duffield et al. 2011). NSIs encompass a range of patient outcomes, including mortality, FTR, readmissions, HAIs such as pneumonia, sepsis, CAUTI and CLABSI, falls and HAPUs (Aiken, Cimiotti, et al. 2011; L. Aiken et al. 2014; Ball et al. 2018; Bettencourt et al. 2020; Daus et al. 2024; Esparza et al. 2012; Fagerström et al. 2018; Frith et al. 2010; He et al. 2016; Kendall‐Gallagher et al. 2011; Kumar et al. 2022; Lasater and Mchugh 2016; Ma et al. 2015; McHugh et al. 2016; Meredith et al. 2024; Needleman et al. 2011; Peutere et al. 2023; Porcel‐Gálvez et al. 2020; Schlak et al. 2021; Shang et al. 2019; Staggs et al. 2012, 2017; Trinkof et al. 2011; Wieczorek‐Wojcik et al. 2020). The NWE influences the following outcomes: higher nursing staffing levels are linked with reduced patient mortality, fewer readmissions, lower rates of HAIs, falls and pressure ulcers and shorter LOS (Aiken, Cimiotti, et al. 2011; Bettencourt et al. 2020; Catania et al. 2024; Esparza et al. 2012; Fagerström et al. 2018; Frith et al. 2010; He et al. 2016; Kendall‐Gallagher et al. 2011; Lasater, McHugh, et al. 2021b; Lasater, McHugh, et al. 2021a; Lasater, Sloane, et al. 2021; Lasater and Mchugh 2016; Ma et al. 2015; McHugh et al. 2016; Meredith et al. 2024; Needleman et al. 2011; Peutere et al. 2024; Schlak et al. 2021; Shang et al. 2019; Staggs et al. 2012; Trinkof et al. 2011; Wieczorek‐Wojcik et al. 2020), whereas nurse understaffing or high workload increases the risk of adverse events and mortality (Catania et al. 2024; Fagerström et al. 2018; Fasoi et al. 2020; Margadant et al. 2020; Peutere et al. 2024). Furthermore, a higher proportion of BSN‐prepared nurses is linked to lower patient mortality and FTR (Aiken, Cimiotti, et al. 2011; L. Aiken et al. 2014; Ball et al. 2018; Haegdorens et al. 2019; Harrison et al. 2019; Kendall‐Gallagher et al. 2011; Kumar et al. 2022; Rochefort et al. 2022; Rochefort et al. 2020), and better nurse work environments lead to lower mortality, FTR and readmissions (Aiken, Cimiotti, et al. 2011; Bettencourt et al. 2020; Daus et al. 2024; Lasater and Mchugh 2016; McHugh et al. 2016; Olds et al. 2017; Rao et al. 2017; Schlak et al. 2021).

##### 4.3.2.6 Enabling Features of the Nursing Work Environment

Studies identified features of the NWE that enabled nurses to maintain patient safety despite challenging conditions. Collegial support, teamwork and a positive safety climate were described as factors that mitigated safety risks and were linked to lower mortality and fewer adverse events (Dubois et al. 2013; Ma et al. 2015; Olds et al. 2017; Salvador et al. 2021). Clinical experience, nurse autonomy and professional judgement were further identified as enabling factors. Higher proportions of experienced and BSN‐prepared nurses, greater nursing autonomy and continuity of staffing were linked to reduced mortality, lower FTR rates and fewer complications (Ambrosi et al. 2017; Haegdorens et al. 2019; Rao et al. 2017; Rochefort et al. 2020). Studies also highlighted the role of organisational and managerial support. Improvements in staffing levels were shown to have the greatest impact on patient outcomes in hospitals with positive work environments, which also buffered the negative effects of nurse burnout on patient safety outcomes (Aiken, Cimiotti, et al. 2011; Bettencourt et al. 2020; Schlak et al. 2021).

Figure 2 sums up the relationship of the key factors relevant to the relationship between NWEs and patient safety. In line with JBI, the figure presents a descriptive data‐mapping of relationships across the studies; it does not imply causal associations. Elements such as staffing levels, work environment conditions, nurse education, organisational culture, patient complexity and missed care are described as interconnected in ways that influence each other. The figure represents pathways described as contributing to either increased risks or more supportive relationships. For example, low staffing leads to an increased workload, missed care and potential safety concerns. On the other hand, adequate staffing, education or strong leadership may be linked to improved care quality and safety. Patient complexity, particularly in the context of multimorbidity, is described as increasing care demands. In resource‐constrained settings, this may contribute to missed care, whereas positive environments and education may help mitigate risks.

**FIGURE 2:**
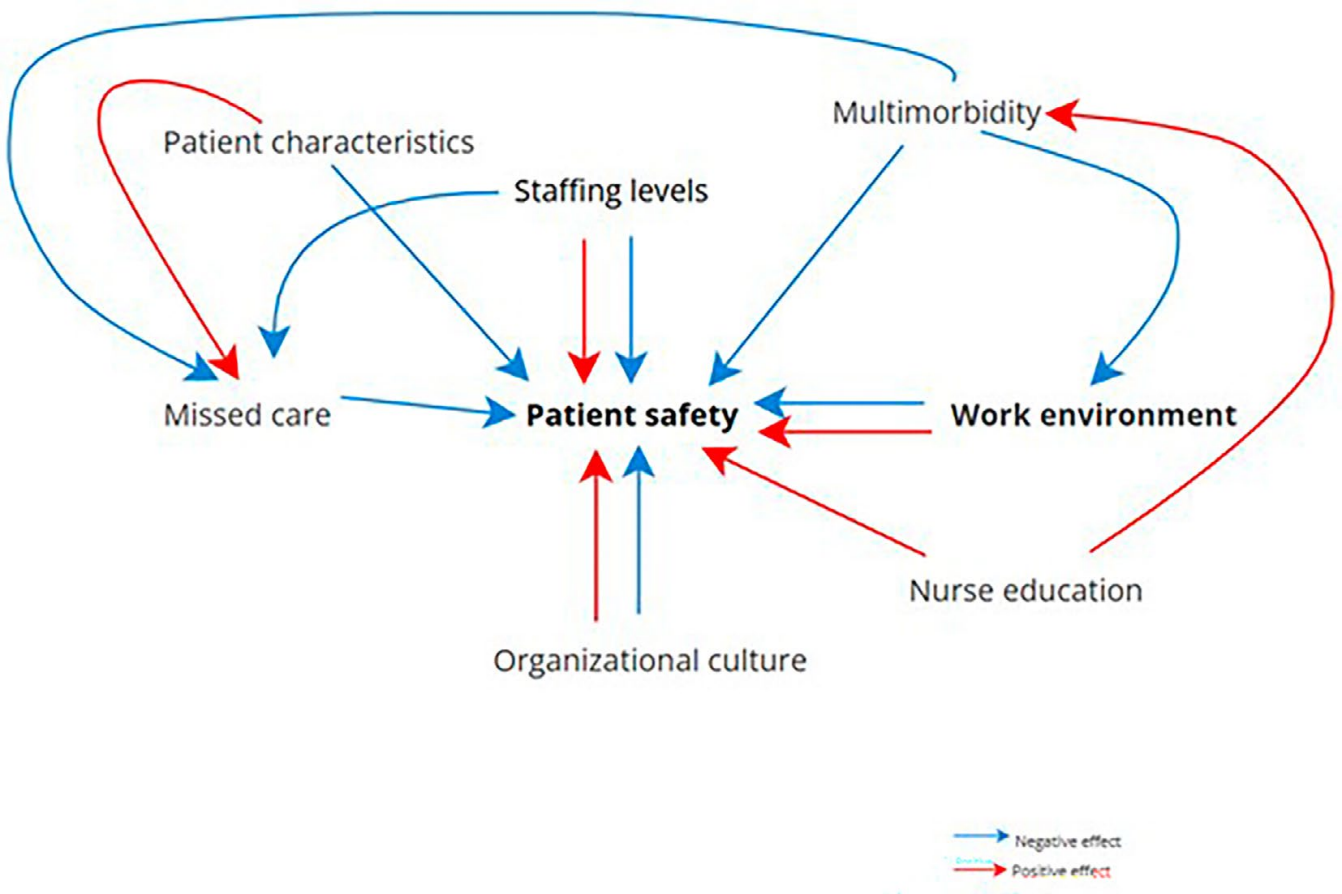
Loop diagram of factors impacting the work environment–patient safety relationship.

Figure 2 provides an overview based on a synthesis of quantitative studies. Building on this, the inclusion of knowledge users allows the findings to be interpreted in a practical context, which can highlight dynamics that may not be captured in quantitative data. This approach is informed by participatory principles and aims to bridge evidence synthesis and real‐world relevance.

#### 4.3.3 Insights From Knowledge Users

The knowledge users emphasised that predictability, skill mix and onboarding processes impact the ability to provide safe care. They reported that flexibility in scheduling, access to sparring partners and a sense of staff safety influence the ability to manage complex patients. Good working conditions were considered a prerequisite for maintaining vigilance, responding to confusion states and avoiding missed care. Some linked poor structural support to increased risks, such as readmission, dehydration and falls. The knowledge users highlighted that educational level, professional development and clinical experience contribute to safer care and a stronger sense of self‐efficacy, professional pride and meaningful work. Knowledge sharing and curiosity were described as essential for confidence in decision‐making.

Leadership emerged as central. The knowledge users highlighted managerial support, trust and a clear sense of direction and structure in their daily work. Unclear expectations were seen as a barrier to effective decision‐making. The knowledge users pointed out the importance of psychological safety and trust as foundational factors. Collegial support, collaboration and a shared sense of well‐being were described as essential. Conversely, unclear expectations strained the work environment by causing unpredictability and therefore a risk to patients' safety. The role of family members was seen as important in ensuring safety. The knowledge users noted that exhaustion of family members can become a safety concern in terms of the family's ability to participate as well as emotional strain. Readmissions, uncertainty and lack of support can leave families overwhelmed.

The knowledge users emphasised clinical risks observed in practice, including aggression, belt restraint and confusion states, as well as physical risks such as falls, pressure ulcers and dehydration. They also identified medication errors, anxiety and readmission as indicators of care complexity.

## 5 Discussion

This review aimed to identify and map the existing literature on the relationship between NWEs and patient safety for patients aged 65 and over. We provided an overview of 84 studies exploring NWEs and patient safety, including the relationship and the theoretical frameworks underpinning them. This was done in collaboration with knowledge users to ensure that multiple perspectives and insights were included.

Based on the findings of this review, the relationship between NWEs and patient safety may be characterised as a wicked problem (Thomas et al. 2018). A wicked problem is a problem that is inherently complex, hard to define and impossible to solve with a single, straightforward solution. Our findings illustrate this complexity: staffing levels, skill mix, multimorbidity and organisational culture are intertwined, and no single factor deterministically predicts patient safety outcomes. Improvements in one area may shift risks elsewhere. For example, increasing nursing staffing may reduce mortality but may also require changes in skill mix, workflow or interprofessional relationships. In the same way, addressing burnout cannot be done in isolation from structural factors such as workload or resource allocation.

The lack of standard definitions for significant terms such as NWEs may impact interpretability and lead to inconsistency and varied conclusions. Standardised definitions are essential for identifying what metrics are relevant in supporting targeted and effective interventions. The NWE has been defined in various ways by different organisations. The International Centre for Human Resources in Nursing described a positive practice environment as *settings supporting excellence and decent work* (International Centre for Human Resources in Nursing, n.d.), the American Nurses Association described it as *safe*, *empowering*, *and satisfying* (Healthy Work Environment for Nurses|ANA Enterprise 2017), whereas the American Association of Critical‐Care Nurses used six standards: *skilled communication*, *true collaboration*, *effective decision‐making*, *appropriate staffing*, *meaningful recognition and authentic leadership* (American Association of Critical‐Care Nurses, n.d.). However, despite the centrality of these definitions, only a minority of the studies included in this review explicitly define them, which may limit the ability to standardise interventions, particularly across healthcare settings. Some studies focus on specific aspects such as nurse‐to‐patient ratios, whereas others emphasise dynamics such as communication and collaboration, which results in varying conclusions about what constitutes the optimal work environment. This ambiguity may obscure which elements are most influential for patient safety outcomes.

When interpreted through contemporary patient safety perspectives, the findings extend beyond an understanding of patient safety as the absence of harm. Several NWE features can be understood as safety‐enabling conditions rather than solely as risk factors when absent. This aligns with Safety‐II, which conceptualises patient safety as emerging from everyday performance within complex healthcare systems. From this perspective, positive and effective work environments support nurses' capacity to anticipate clinical risks, make adjustments under varying workloads and maintain safe care, thereby complementing traditional Safety‐I approaches.

Staffing emerged as a prominent subject, and although reliance on temporary staff can provide short‐term relief, it is often ineffective in sustaining care quality (Tamburello et al., n.d.). Increasing baseline staffing and introducing standardised staffing policies can lead to improvements in care quality and reductions in mortality, LOS and readmissions, as well as to cost savings in the long run (McHugh et al. 2021). However, Halm (2019) argues that improvements in staffing are insufficient without attention to the broader practice environment. To address this, current and future nurses should be prepared with the necessary knowledge, including by integrating safety and quality improvement competencies into nursing curricula (Zaitoun et al. 2023), to ensure that professionals are adequately equipped to maintain safe and high‐quality care. Furthermore, staff retention and continuity in care must be considered (Sandvik and Hunskaar 2024), as high workloads contribute to nurse burnout, which further compromises patient safety. In Garcia et al.'s (2019) systematic review, burnout was directly linked to deteriorating patient safety, which emphasises the need for improving the workload balance and emotional support. Similarly, MacPhee et al. (2017) found that heavy workloads impact both patient and nurse outcomes, which highlights the need to focus on proactive strategies to offer a sustainable approach to maintaining high standards, efficiency and reducing financial strains.

A limited number of studies indicate that multimorbidity is an important yet inconsistently addressed factor in understanding patient safety. Research focusing on patients with coexisting physical and mental health conditions highlights the need for coordination between multiple specialties, which may increase fragmented care, missed care and adverse events (Doherty and Gaughran 2014). As of now, the studies frequently rely on comorbidity indices; however, these tools rarely capture outcomes specific to patients experiencing both mental and physical illness. Mental health conditions are known to exacerbate physical illness outcomes (Formánek et al. 2024), and relying on a broad comorbidity score risks aggregating multiple diagnoses under a general ‘burden’, which may lead to a lack of insight into the specific vulnerabilities. Across studies, the complexity of multimorbidity is described as requiring advanced nursing skills and coordination; nevertheless, such complexity is not consistently addressed in general patient safety literature (Brickell and McLean 2011). This may limit nurses' capacity to provide safe care, although this relationship has not been systematically explored.

Older patients face heightened risks due to chronic conditions, frailty, polypharmacy and age‐related decline in physiological functions, all of which increase the risk of safety breaches (Alotaibi et al. 2025). Elderly patients are more likely to experience longer hospital stays and admissions across settings, and they are naturally at a greater risk for falls, pressure ulcers and mortality. However, most of the studies did not delineate the age group of the patients, and some did not specify the specific outcomes for the elderly population. This limits the ability to determine whether the findings are fully applicable to patients aged 65 and over, as only 13 studies specifically targeted this group.

Given the distinct age‐related vulnerabilities of elderly patients, it is plausible that patient safety outcomes would differ in a younger population. Studies conducted within paediatric care report different outcomes, such as peripheral intravenous infiltrates, fluid overload, conjunctivitis (Wilson et al. 2011) and communication breakdowns (Lake et al. 2021), which differ from the outcomes observed in this review. These observations highlight a gap in the literature regarding age‐specific reporting. However, the underlying NWE mechanisms appear consistent across age groups. Core mechanisms such as staffing adequacy, teamwork and leadership responsiveness are cross‐cutting determinants of safety. What differs are the population‐specific manifestations in harm, for example, falls in older adults, which arise due to age‐related multimorbidity, cognitive vulnerability and functional decline rather than fundamentally different work environment dynamics.

Across studies, the application of theoretical frameworks was heterogeneous. Some relied on quality improvement models or organisational frameworks, which tend to be practical or system‐oriented rather than theoretically grounded. These were used descriptively to structure variables rather than to generate deeper theoretical explanations. Several frameworks are dated or developed for narrow organisational contexts, which limit transferability to contemporary hospital settings or to the complexities of caring for older adults. This suggests that the field may be under‐theorised, with limited uptake of more recent or comprehensive theoretical perspectives. Our synthesis highlights a need for future research to apply more contemporary theory to better explain mechanisms linking NWEs and patient safety outcomes for older adults.

Another research gap concerns the theme of *mental health care settings*, as studies within these environments are underrepresented. Challenges such as workplace violence are a prominent issue in mental health care (Mento et al. 2020); notwithstanding, the impact of this on patient safety has not been adequately addressed. Coercive measures create complex working conditions, thereby compromising care quality (Gustafsson and Salzmann‐Erikson 2016). For example, Roche et al. (2010) found that 9% of all patient safety‐related incidents involved patients and physical violence or violent verbal exchange, which underscores the need for targeted strategies to mitigate violence and its effects on safety and staff well‐being. Nevertheless, patient safety within mental health care remains underexplored, although research has emphasised the need for greater awareness and education in this area (Brickell and McLean 2011). Similarly, Kanerva et al. (2013) have highlighted the limited focus on these environments and pointed to the definition of a safety culture as an important element in creating safe practices.

In Brickell and McLean's (2011) qualitative analysis, specific adverse events in mental health care, including suicide, self‐harm and harm from seclusion and restraint use, are highlighted. Importantly, the present review only included quantitative studies and therefore did not capture the experiential nuances that qualitative research can provide. Qualitative studies, such as Brickell and McLean's, offer complementary perspectives that are not visible in quantitative data. Other research has explored outcomes, such as absconding (Ngune et al. 2023), self‐injuries/self‐harm (Ngune et al. 2023), disruptive and threatening behaviour (Beckman et al. 2022), arson (Boumans et al. 2015), coercive measures, such as seclusion (Doedens et al. 2017) and mechanical restraint (Kodal et al. 2018). Skill, adequate training, education, team performance and patient‐centredness are considered prerequisites for patient safety, and staff performance and competence as factors to achieve safer psychiatry (Svensson 2022). However, further questions remain regarding how interventions and theory can be integrated into practice, particularly in mental health settings, due to a perhaps more intricate relationship between staffing levels, skill mix and outcomes than in somatic care, where an increase in staff often reduces adverse events. This suggests a need for outcome measures that are specific to mental health settings.

Recently, efforts to develop such tailored measures have emerged. Desmet et al. (2021) have introduced the Mental Health Nurse‐Sensitive Patient Outcome Scale, which captures patient‐reported outcomes attributable to nursing care. Unlike traditional NSIs, this scale evaluates patients' experiences of nurse–patient relationships, empowerment, communication and contextual aspects of care (Desmet et al. 2023). Similarly, Ngune et al. (2023) examined the relationship between nurse variables and patient outcomes in mental health care and found seven relevant nurse‐sensitive outcomes; however, they highlight the need for more research and development. In other words, despite the widespread use of NSIs, reviews report varying ways of measuring patient, nurse and organisational outcomes, which indicates a lack of coherence and consistency (Afaneh et al. 2021; Oner et al. 2021). This underscores the need for clearer conceptual alignment and greater methodological standardisation.

The diverse patient populations, including the elderly and people with multimorbidity, amplify the unpredictability and interdependence of outcomes. Standardised measures of patient safety may fail to capture such vulnerabilities, which highlights that traditional linear problem‐solving processes are inadequate. Addressing this wicked problem requires ongoing monitoring and integration of multiple perspectives rather than seeking a single, definitive solution (McAllister and Brien 2020). By framing our findings within this perspective, we are emphasising why patient safety challenges persist and why future strategies must account for the adaptive requirements inherent in NWEs.

### 5.1 Limitations

Several limitations of this review should be considered. First, the studies encompassed various methodologies, populations and measurement tools, which may have introduced inconsistencies or limited comparability. The exclusion of subjective measures, such as patient satisfaction and nurses' perceptions, improved consistency but may have reduced attention to patient‐centred outcomes and broader aspects of safety.

Although the search strategy included qualitative, quantitative and mixed‐method studies, only quantitative studies met the inclusion criteria. This may have limited the review's ability to capture the relational dimensions, which are often revealed through qualitative inquiry.

Restricting inclusion to studies published after 2010 may have excluded important historical perspectives on the development of NWEs and patient safety. In addition, not all studies applied a uniform age threshold corresponding to exactly 65 years; however, this age cut‐off was used as a reference point to reflect a commonly applied demarcation of older adults in research and clinical practice. This reflects a widely used international definition but may not align with all national classifications or with more nuanced approaches to ageing.

Finally, the involvement of knowledge users enhanced practical relevance but may have shifted the review's focus toward certain priorities, potentially narrowing the breadth of exploration compared with if it had been conducted by the research team alone.

### 5.2 Recommendations for Practice

The findings suggest that attention to staffing adequacy, leadership support and psychosocial work environment factors may be relevant for patient safety in the care of older adults. Healthcare organisations may benefit from monitoring these factors and considering them in quality and safety initiatives. Given the complexity of the matter, locally adapted approaches involving frontline staff are most likely necessary. Furthermore, practice should focus on promoting positive and psychologically safe environments, supporting nurses' well‐being and providing ongoing education for managing complex patient care. Efforts should aim to retain staff and maintain continuity.

## 6 Conclusion

The relationship between NWEs and patient safety for older patients is inconsistently defined across somatic and mental health settings. NWEs were conceptualised through heterogeneous and overlapping dimensions, whereas patient safety was primarily defined implicitly through outcome measures. When core work environment conditions deteriorate, both patients and nurses face increased risks of compromised care quality, safety incidents and declining well‐being. Mapping NSIs across settings provides a foundation for refining how this relationship is understood and measured. Increased awareness may support nurses and policymakers in preventing adverse events related to poor working conditions. Future research should address the intricate nature of the relationship between NWEs and patient safety, an issue which increasingly resembles a wicked problem and which therefore requires integrated and context‐sensitive approaches.

## Author Contributions

T.V.: conceptualisation of the study, in charge of the manuscript writing and preparation, screening, data extraction and data mapping. P.K.S.: screening, data extraction and manuscript feedback. L.H.H.: manuscript feedback. L.M.T.: conceptualisation of the study, assisted with the article search, screening and data extraction. B.H.‐H.: conceptualisation of the study, manuscript feedback, idea generation and a key role in assembling the knowledge user group. L.L.B.: conceptualisation of the study, comprehensive manuscript feedback, rigorous revisions, screening, provided input during the concept development stage and assembled the knowledge user group. All authors have read and approved the final manuscript.

## Funding

The review received funding from MVU‐Fællespuljen (grant number 113512) and the Lån & Spar Fund.

## Disclosure

Permissions: Data utilised in the submitted manuscript have been lawfully acquired in accordance with The Nagoya Protocol on Access to Genetic Resources and the Fair and Equitable Sharing of Benefits Arising from Their Utilization to the Convention on Biological Diversity. The relevant fieldwork permission was obtained from Region Zealand Privacy Research (p‐2024‐173745), Region Zealand research registry REG‐171‐2021, the Danish Research Ethics Committee document ID: 11949188.

PROSPERO: The PROSPERO database identified one ongoing systematic review protocol on the topic from 2016 (https://www.crd.york.ac.uk/PROSPERO/view/36126), but no later publications were found.

Protocol Registration: Open Science Framework: https://osf.io/247uc, https://doi.org/10.17605/OSF.IO/247UC.

## Conflicts of Interest

The authors declare no conflicts of interest.

## Supporting information


**Data S1:** Characteristics and key findings of the included studies.

**Data S2:** Search strategy.

**Data S3:** Elaboration and overview of activities involving knowledge users in the scoping review.

## Data Availability

The data that supports the findings of this study are available in the Supporting Information of this article.
