# Supplementary material for: Nursing Work Environments and Patient Safety in Older Adults: A Scoping Review of Their Intricate Relationship, Definitions and Theoretical Frameworks
Source: J Adv Nurs. 2026 Jul 29;82(12):10171–93. doi: 10.1111/jan.70683 (PMC13752672; doi:10.1111/jan.70683)
Supplement: Supplementary file 1 [file JAN-82-10171-s001.docx]

**Search strategy**

**MEDLINE (PubMed) search conducted on the 22^nd^ of October 2022, the 27^th^ of November 2024 and the 21^st^ of April 2026**

| **PubMed** | | | | |
| --- | --- | --- | --- | --- |
| **#** | **Search** | **Results**   *October 22, 2022* | **Results**   *November 27, 2024* | **Results** *April 21, 2026* |
| 1 | ("Nurses"[MeSH Terms] OR "Nursing Staff"[MeSH Terms] OR "Nursing"[MeSH Terms] OR "Nurses"[Text Word] OR "Nursing Staff"[Text Word] OR "registered nurse"[Text Word]) |  | 528,166 | 552,622 |
| 2 | (((((((((((((("Workplace"[Mesh]) OR "Job Satisfaction"[Mesh]) OR "Personnel Turnover"[Mesh]) OR "Work Engagement"[Mesh]) OR "Workload"[Mesh]) OR "Workplace Violence"[Mesh]) OR "Sick Leave"[Mesh]) OR ( "Occupational Health"[Mesh] OR "Occupational Health Nursing"[Mesh] )) OR "Occupational Stress"[Mesh]) OR "Accidents, Occupational"[Mesh]) OR "Occupational Injuries"[Mesh]) OR "Shift Work Schedule"[Mesh]) OR "Work-Life Balance"[Mesh]) OR ((((((((((((((((((((work environment) OR (working environment)) OR (workplace environment)) OR (workspace environment)) OR ("work climate")) OR ("working climate")) OR ("work motivation")) OR ("work experience")) OR ("nurse turnover")) OR ("nurse shortage")) OR ("nursing shortages")) OR (intent-to-leave)) OR ("nurse-patient" ratio)) OR (nurse-to-patient ratio)) OR (minimum nurse-to-patient ratio)) OR (understaffing)) OR ("nurse retention")) OR ("nurse recruitment")) OR ("temporary employment")) OR ("quality of working life"))) |  | 380,911 | 436,990 |
| 3 | ((((((("Hospitals"[Mesh]) OR "Hospital Units"[Mesh]) OR "Mental Health"[Mesh]) OR ( "Ambulatory Care"[Mesh] OR "Ambulatory Care Facilities"[Mesh] )) OR "Inpatients"[Mesh]) OR "Outpatients"[Mesh]) OR ("regional health service")) |  | 672,098 | 704,292 |
| 4 | ((((((((((((((("Patient Safety"[Mesh]) OR "Safety Management"[Mesh]) OR "Patient Harm"[Mesh]) OR "Hospital Mortality"[Mesh]) OR "Medical Errors"[Mesh]) OR "Cross Infection"[Mesh]) OR "Patient Readmission"[Mesh]) OR "Violence"[Mesh]) OR "Coercion"[Mesh]) OR "Aggression"[Mesh]) OR "Restraint, Physical"[Mesh]) OR "Self-Injurious Behavior"[Mesh]) OR "Outcome and Process Assessment, Health Care"[Mesh]) OR "Quality Indicators, Health Care"[Mesh]) OR (((((((("patient safety") OR ("adverse events")) OR (coercion)) OR (seclusion)) OR (self-harm)) OR (nurse-sensitive indicators)) OR (nurse-sensitive outcome)) OR ("nursing outcomes"))) |  | 2,178,962 | 2,335,788 |
| 5 | #1 AND #2 AND #3 AND #4 | 1474 | 1519 | 1,682 |
| 6 | Filters: Danish, English, Norwegian, Swedish | 1408 | 1448 | 1,608 |
| 7 | Search: ("2022/10/25"[Date - Publication] : "2024/11/27"[Date - Publication]) Filters: Danish, English, Norwegian, Swedish |  | 3,300,932 |  |
|  | Seach: (2024/11/27”[Date - Publication] : "2026/04/21"[Date - Publication]) Filters: Danish, English, Norwegian, Swedish |  |  | 2,498,706 |
| 8 | Search: #1 AND #2 AND #3 AND #4 AND #6 AND #7 Filters: Danish, English, Norwegian, Swedish |  | 159 | 152 |

**The search was performed using "topic" because "all fields" returned too many results (over 10,000)*

**Web of Science (Clarivate) search conducted on the 22^nd^ of October 2022, the 27^th^ of November 2024 and the 21^st^ of April 2026**

| **Cinahl** | | | | |
| --- | --- | --- | --- | --- |
| **#** | **Search** | **Results**   *October 22, 2022* | **Results**   *November 27, 2024* | **Results** *April 21, 2026* |
| 1 | (((TS=(nurs*)) OR TS=(nursing staff)) OR TS=(registered nurse)) OR TS=(nurse practitioner*) | 276,864 | 409,679 | 803,535 |
| 2 | (((((((((((((((((((((((((TS=("work* environment")) OR TS=("work engagement")) OR TS=("work motivation")) OR TS=("work experience")) OR TS=("job satisfaction")) OR TS=("psychological safety")) OR TS=("psychological security")) OR TS=(workload)) OR TS=(stress)) OR TS=(burnout)) OR TS=("workrelated injuries")) OR TS=("workrelated accidents")) OR TS=("workrelated violence")) OR TS=(turnover)) OR TS=("nurse shortage")) OR TS=("personnel shortage")) OR TS=("nurse-patient ratio")) OR TS=("minimum nurse-to-patient ratio")) OR TS=("sick leave")) OR TS=("intent to leave")) OR TS=(retention)) OR TS=(recruitment)) OR TS=(understaffing)) OR TS=("shift work")) OR TS=("work life balance")) OR TS=("quality of work* life") | 2,377,394 | 3,183,002 | 4,988,718 |
| 3 | (((((((((((((((((((((((((TS=("patient safety")) OR TS=("patient harm")) OR TS=("risk management")) OR TS=("safety management")) OR TS=("adverse health care events")) OR TS=("adverse events")) OR TS=("hospital mortality")) OR TS=("cross infections")) OR TS=(readmission)) OR TS=(violence)) OR TS=(coercion)) OR TS=(seclusion)) OR TS=(aggression)) OR TS=("aggressive behavior")) OR TS=("patient assault")) OR TS=(restraint)) OR TS=("physical restraint")) OR TS=("self-injuries behavior")) OR TS=(suicide)) OR TS=(self-harm)) OR TS=(outcome)) OR TS=("outcome assessment")) OR TS=("nursing outcome*")) OR TS=("nurse sensitive indicators")) OR TS=("nurse sensitive outcome*")) OR TS=("quality indicators") | 2,736,035 | 3,882,642 | 4,437,205 |
| 4 | ((((((((((((TS=(general hospital*)) OR TS=(special hospital*)) OR TS=(public hospital*)) OR TS=(private hospital*)) OR TS=(magnet hospital*)) OR TS=(psychiatric hospital*)) OR TS=("hospital unit*")) OR TS=(inpatients)) OR TS=(outpatients)) OR TS=("ambulatory care")) OR TS=("regional health service")) OR TS=("clinical settings")) OR TS=("health facilities") | 426,089 | 620,828 | 553,642 |
| 5 | #4 AND #3 AND #2 AND #1 | 2,102 | 3,206 | 740 |
| 6 | #4 AND #3 AND #2 AND #1 and Danish or English (Languages) | 2,055 | 721 | 733 |
|  | Timespan: 2024-11-27 to 2026-04-21 |  |  | 682 |

**Embase (OVID) search conducted on the 22^nd^ of October 2022, the 27^th^ of November 2024 and the 21^st^ of April 2026**

| **Embase** | | | | |
| --- | --- | --- | --- | --- |
| **#** | **Search** | **Results**   *October 22, 2022* | **Results**   *November 27, 2024* | **Results** *April 21, 2026* |
| 1 | nurse practitioner/ or exp nurse/ or registered nurse/ | 206,595 | 231,525 | 258,976 |
| 2 | exp nursing/ or nursing staff/ | 421,605 | 438,738 | 458,154 |
| 3 | 1 or 2 | 579,971 | 618,040 | 660,186 |
| 4 | exp work environment/ | 34,177 | 40,655 | 45,656 |
| 5 | exp organizational climate/ | 397 | 466 | 493 |
| 6 | exp job satisfaction/ | 34,105 | 38,143 | 41,642 |
| 7 | exp nurse patient ratio/ | 414 | 463 | 513 |
| 8 | intent-to-leave.mp. | 403 | 521 | 644 |
| 9 | minimum nurse-to-patient ratios.mp. | 24 | 26 | 27 |
| 10 | exp professional well-being/ | 287 | 685 | 985 |
| 11 | exp personnel management/ | 96,988 | 103,485 | 108,933 |
| 12 | exp nursing shortage/ | 455 | 629 | 758 |
| 13 | exp personnel shortage/ | 3,464 | 5,082 | 6,655 |
| 14 | exp workload/ | 51,245 | 58,461 | 67,556 |
| 15 | exp work engagement/ | 2,270 | 3,237 | 4,468 |
| 16 | exp work experience/ | 12,134 | 14,890 | 16,987 |
| 17 | exp nurse patient ratio/ | 414 | 463 | 513 |
| 18 | exp occupational health/ | 250,476 | 271,625 | 293,894 |
| 19 | exp occupational accident/ | 24,890 | 26,501 | 28,999 |
| 20 | exp job stress/ | 14,107 | 17,809 | 27,145 |
| 21 | exp burnout/ | 26,411 | 34,945 | 42,480 |
| 22 | exp workplace violence/ | 2,096 | 2,696 | 3,270 |
| 23 | exp work-life balance/ | 2,541 | 3,673 | 4,742 |
| 24 | exp employment status/ | 43,133 | 53,247 | 61,628 |
| 25 | 4 or 5 or 6 or 7 or 8 or 9 or 10 or 11 or 12 or 13 or 14 or 15 or 16 or 17 or 18 or 19 or 20 or 21 or 22 or 23 or 24 | 467,501 | 523,387 | 574,733 |
| 26 | nurse practitioner/ or exp nurse/ or registered nurse/ or exp nursing/ or nursing staff/ | 579,971 | 618,040 | 660,186 |
| 27 | exp work environment/ or exp organizational climate/ or exp job satisfaction/ or exp nurse patient ratio/ or intent-to-leave.mp. or minimum nurse-to-patient ratios.mp. or exp professional well-being/ or exp personnel management/ or exp nursing shortage/ or exp personnel shortage/ or exp workload/ or exp work engagement/ or exp work experience/ or exp nurse patient ratio/ or exp occupational health/ or exp occupational accident/ or exp job stress/ or exp burnout/ or exp workplace violence/ or exp work-life balance/ or exp employment status/ | 467,501 | 523,387 | 574,733 |
| 28 | exp hospital/ | 1,330,054 | 1,531,089 | 1,714,562 |
| 29 | exp mental health/ | 209,813 | 274,345 | 356,679 |
| 30 | exp ambulatory care/ | 52,350 | 56,041 | 59,459 |
| 31 | exp outpatient care/ | 44,052 | 51,222 | 57,044 |
| 32 | exp hospital patient/ | 222,543 | 253,000 | 262,078 |
| 33 | exp outpatient/ or exp outpatient department/ | 225,038 | 262,866 | 296,333 |
| 34 | regional health service.mp. | 337 | 393 | 448 |
| 35 | inpatient.mp. | 173,009 | 206,676 | 238,036 |
| 36 | 28 or 29 or 30 or 31 or 32 or 33 or 34 or 35 | 1,875,000 | 2,189,201 | 2,487,933 |
| 37 | exp hospital/ or exp mental health/ or exp ambulatory care/ or exp outpatient care/ or exp hospital patient/ or exp outpatient/ or exp outpatient department/ or regional health service.mp. or inpatient.mp. | 1,875,000 | 2,189,201 | 2,487,933 |
| 38 | exp patient safety/ | 153,904 | 180,776 | 20,4458 |
| 39 | exp hospital mortality/ | 56,544 | 77,275 | 87,831 |
| 40 | exp adverse event/ | 737,292 | 1,062,720 | 1,327,417 |
| 41 | exp adverse outcome/ | 62,445 | 74,547 | 84,361 |
| 42 | exp medical error/ | 166,374 | 193,374 | 224,479 |
| 43 | exp patient harm/ | 2,534 | 3,342 | 4,134 |
| 44 | exp cross infection/ | 20,362 | 21,706 | 22,719 |
| 45 | exp hospital readmission/ | 88,762 | 113,402 | 134,299 |
| 46 | violence/ | 51,621 | 56,449 | 59,410 |
| 47 | exp coercion/ | 312 | 849 | 1,445 |
| 48 | exp aggression/ | 120,652 | 143,870 | 158,340 |
| 49 | exp physical restraint/ | 610 | 1,199 | 1,804 |
| 50 | exp automutilation/ | 22,353 | 27,296 | 31,927 |
| 51 | suicidal behavior/ | 17,816 | 22,122 | 26,235 |
| 52 | outcome assessment/ | 742,246 | 913,182 | 984,505 |
| 53 | health care quality/ | 263,236 | 285,792 | 301,889 |
| 54 | exp nursing outcome/ | 110 | 128 | 156 |
| 55 | exp patient safety indicator/ | 68 | 100 | 130 |
| 56 | exp "quality of nursing care"/ | 505 | 593 | 687 |
| 57 | treatment outcome/ | 937,630 | 1,023,849 | 1,108,603 |
| 58 | clinical outcome/ | 259,991 | 398,531 | 540,009 |
| 59 | safety management.mp. | 3,107 | 3,848 | 4,649 |
| 60 | seclusion.mp. | 1,818 | 2,062 | 2,239 |
| 61 | nurse-sensitive indicators.mp. | 54 | 66 | 77 |
| 62 | nurse-sensitive outcome.mp. | 23 | 26 | 27 |
| 63 | 38 or 39 or 40 or 41 or 42 or 43 or 44 or 45 or 46 or 47 or 48 or 49 or 50 or 51 or 52 or 53 or 54 or 55 or 56 or 57 or 58 or 59 or 60 or 61 or 62 | 3,274,479 | 4,009,864 | 4,598,356 |
| 64 | exp patient safety/ or exp hospital mortality/ or exp adverse event/ or exp adverse outcome/ or exp medical error/ or exp patient harm/ or exp cross infection/ or exp hospital readmission/ or violence/ or exp coercion/ or exp aggression/ or exp physical restraint/ or exp automutilation/ or suicidal behavior/ or outcome assessment/ or health care quality/ or exp nursing outcome/ or exp patient safety indicator/ or exp "quality of nursing care"/ or treatment outcome/ or clinical outcome/ or safety management.mp. or seclusion.mp. or nurse-sensitive indicators.mp. or nurse-sensitive outcome.mp. | 3,274,479 | 4,009,864 | 4,598,356 |
| 65 | 26 and 27 and 37 and 64 | 2,984 | 3,676 | 4,521 |
| 66 | Limit 65 to (danish or english or norwegian or swedish) |  | 3,545 | 4,378 |
| 67 | Limit 66 to dd=20221025-20241127 |  | 281 |  |
|  | Limit 66 to dd=20241127-20260421 |  |  | 804 |

**APA PsycINFO (OVID) search conducted on the 22^nd^ of October 2022, the 27^th^ of November 2024 and the 21^st^ of April 2026**

| **APA PsychInfo** | | | | |
| --- | --- | --- | --- | --- |
| **#** | **Search** | **Results**  *October 22, 2022* | **Results**  *November 27, 2024* | **Results** *April 21, 2026* |
| 1 | nurses.mp. or exp Nurses/ | 65,737 | 74,517 | 79,687 |
| 2 | exp Nursing/ | 25,652 | 29,153 | 31,178 |
| 3 | nursing staff.mp. | 9,793 | 10,501 | 10,956 |
| 4 | registered nurse.mp. | 1,326 | 1,501 | 1,584 |
| 5 | nurse practitioners.mp. | 2,861 | 3,395 | 3,613 |
| 6 | 1 or 2 or 3 or 4 or 5 | 78,232 | 88,458 | 94,251 |
| 7 | nurses.mp. or exp Nurses/ or exp Nursing/ or nursing staff.mp. or nurse practitioners.mp. or registered nurse.mp. | 78,232 | 88,458 | 94,251 |
| 8 | exp Working Conditions/ | 32,219 | 36,354 | 46,118 |
| 9 | exp Organizational Climate/ | 13,068 | 14,155 | 15,032 |
| 10 | exp Job Satisfaction/ | 21,112 | 23,314 | 24,997 |
| 11 | exp Employee Well Being/ | 366 | 989 | 1,860 |
| 12 | exp Employee Engagement/ | 3,700 | 4,992 | 6,114 |
| 13 | exp Employee Turnover/ | 6,050 | 7,279 | 8,331 |
| 14 | exp Employee Motivation/ | 3,412 | 3,888 | 4,300 |
| 15 | exp Work Load/ | 3,406 | 5,941 | 5,846 |
| 16 | exp Job Experience Level/ | 4,049 | 4,520 | 4,951 |
| 17 | exp Occupational Health/ | 5,001 | 6,186 | 5,754 |
| 18 | exp occupational health psychology/ | 197 | 251 | 270 |
| 19 | exp occupational safety/ | 4,136 | 4,753 | 5,160 |
| 20 | exp Occupational Stress/ | 24,740 | 27,297 | 28,672 |
| 21 | exp workplace violence/ | 1,102 | 1,398 | 1,583 |
| 22 | exp Occupational Exposure/ | 1,309 | 1,427 | 1,487 |
| 23 | exp Work Scheduling/ | 1,865 | 2,122 | 2,298 |
| 24 | exp Employee Retention/ | 1,645 | 2,407 | 3,157 |
| 25 | exp Personnel Recruitment/ | 3,310 | 3,825 | 4,136 |
| 26 | exp Employment Status/ | 30,317 | 34,165 | 33,594 |
| 27 | exp Work-Life Balance/ | 1,379 | 1,882 | 2,275 |
| 28 | exp "Quality of Work Life"/ | 1,713 | 1,903 | 2,041 |
| 29 | work* environment.mp. | 11,481 | 13,633 | 15,659 |
| 30 | nurse shortage.mp. | 45 | 54 | 63 |
| 31 | personnel shortage.mp. | 24 | 29 | 30 |
| 32 | intent to leave.mp. | 530 | 610 | 682 |
| 33 | nurse-patient ratio.mp. | 29 | 32 | 33 |
| 34 | nurse to patient ratio.mp. | 60 | 70 | 78 |
| 35 | minimum of nurse to patient ratios.mp. | 2 | 3 | 3 |
| 36 | understaffing.mp. | 127 | 166 | 197 |
| 37 | 8 or 9 or 10 or 11 or 12 or 13 or 14 or 15 or 16 or 17 or 18 or 19 or 20 or 21 or 22 or 23 or 24 or 25 or 26 or 27 or 28 or 29 or 30 or 31 or 32 or 33 or 34 or 35 or 36 | 135,509 | 156,109 | 165,538 |
| 38 | exp Working Conditions/ or exp Organizational Climate/ or exp Job Satisfaction/ or exp Employee Well Being/ or exp Employee Engagement/ or exp Employee Turnover/ or exp Employee Motivation/ or exp Work Load/ or exp Job Experience Level/ or exp Occupational Health/ or exp occupational health psychology/ or exp occupational safety/ or exp Occupational Stress/ or exp workplace violence/ or exp Occupational Exposure/ or exp Work Scheduling/ or exp Employee Retention/ or exp Personnel Recruitment/ or exp Employment Status/ or exp Work-Life Balance/ or exp "Quality of Work Life"/ or work* environment.mp. or nurse shortage.mp. or personnel shortage.mp. or intent to leave.mp. or nurse-patient ratio.mp. or nurse to patient ratio.mp. or minimum of nurse to patient ratios.mp. or understaffing.mp. | 135,509 | 156,109 | 165,538 |
| 39 | exp Hospitals/ | 26,165 | 30,551 | 32,658 |
| 40 | exp Health Care Services/ | 235,454 | 334,100 | 359,016 |
| 41 | exp Mental Health/ | 82,010 | 104,113 | 118,165 |
| 42 | exp Outpatients/ | 7,827 | 8,858 | 9,333 |
| 43 | exp Outpatient Treatment/ | 7,778 | 8,820 | 9,527 |
| 44 | exp Hospitalized Patients/ | 13,901 | 15,399 | 16,398 |
| 45 | ambulatory care.mp. | 10,001 | 10,418 | 10,560 |
| 46 | regional health service.mp. | 30 | 34 | 40 |
| 47 | inpatients.mp. | 23,283 | 31,107 | 31,922 |
| 48 | 39 or 40 or 41 or 42 or 43 or 44 or 45 or 46 or 47 | 367,943 | 486,964 | 526,880 |
| 49 | exp Hospitals/ or exp Hospitalized Patients/ or inpatients.mp. or exp outpatients/ or exp outpatient treatment/ or ambulatory care.mp. or exp mental health/ or exp health care services/ or regional health service.mp. | 367,943 | 486,964 | 526,880 |
| 50 | exp Patient Safety/ | 3,054 | 4,353 | 5,093 |
| 51 | exp Risk Management/ | 10,220 | 11,810 | 12,361 |
| 52 | exp Hospital Admission/ | 5,995 | 6,854 | 7,308 |
| 53 | exp Patient Violence/ | 1,420 | 1,599 | 1,694 |
| 54 | exp Coercion/ | 2,750 | 3,411 | 3,740 |
| 55 | exp Aggressive Behavior/ | 176,230 | 233,130 | 249,357 |
| 56 | exp Physical Restraint/ | 2,289 | 2,479 | 2,586 |
| 57 | exp Treatment Outcomes/ | 133,478 | 162,436 | 181,709 |
| 58 | exp "Quality of Care"/ | 14,556 | 15,769 | 16,504 |
| 59 | exp Health Outcomes/ | 4,556 | 7,931 | 9,970 |
| 60 | exp Self-Destructive Behavior/ | 46,374 | 58,390 | 64,572 |
| 61 | exp Self-Mutilation/ | 1,157 | 8,787 | 9,949 |
| 62 | exp Self-Injurious Behavior/ | 7,310 | 8,787 | 9,949 |
| 63 | safety management.mp. | 2,118 | 2,300 | 2,413 |
| 64 | hospital mortality.mp. | 1,168 | 1,318 | 1,391 |
| 65 | cross infections.mp. | 1 | 1 | 1 |
| 66 | exp Patient Seclusion/ | 592 | 689 | 730 |
| 67 | nurse-sensitive indicators.mp. | 10 | 13 | 12 |
| 68 | nurse-sensitive outcome.mp. | 9 | 10 | 10 |
| 69 | nursing outcomes.mp. | 93 | 104 | 118 |
| 70 | quality indicators.mp. | 2,597 | 2,819 | 2,928 |
| 71 | outcome assessment.mp. | 13,440 | 14,157 | 14,548 |
| 72 | health care quality.mp. | 899 | 987 | 1,035 |
| 73 | exp patient safety/ or exp patient seclusion/ or exp patient violence/ or exp risk management/ or safety management.mp. or patient harm.mp. or hospital mortality.mp. or cross infections.mp. or exp hospital admission/ or exp Coercion/ or exp Aggressive Behavior/ or exp Physical Restraint/ or self-mutilation/ or exp self-injurious behavior/ or exp treatment outcomes/ or patient outcomes.mp. or exp "Quality of Care"/ or Health care Quality.mp. or nurse-sensitive indicators.mp. or nurse-sensitive outcomes.mp. or quality indicators.mp. or exp self-destructive behavior/ or exp health outcomes/ | 391,706 | 494,631 | 539,310 |
| 74 | 50 or 51 or 52 or 53 or 54 or 55 or 56 or 57 or 58 or 59 or 60 or 61 or 62 or 63 or 64 or 65 or 66 or 67 or 68 or 69 or 70 or 71 or 72 | 397,382 | 499,941 | 544,128 |
| 75 | exp Patient Safety/ or exp Risk Management/ or exp Hospital Admission/ or exp Patient Violence/ or exp Coercion/ or exp Aggressive Behavior/ or exp Physical Restraint/ or exp Treatment Outcomes/ or exp "Quality of Care"/ or exp Health Outcomes/ or exp Self-Destructive Behavior/ or exp Self-Mutilation/ or exp Self-Injurious Behavior/ or safety management.mp. or hospital mortality.mp. or cross infections.mp. or exp Patient Seclusion/ or nurse-sensitive indicators.mp. or nurse-sensitive outcome.mp. or nursing outcomes.mp. or quality indicators.mp. or outcome assessment.mp. or health care quality.mp. | 397,382 | 499,941 | 544,128 |
| 76 | 7 and 38 and 49 and 75 | 847 | 1,037 | 1,143 |
| 77 | Limit 76 to (danish or english or norwegian or swedish) |  | 1,016 | 160 |
| 78 | Limit 77 to up=20221025-20241127 |  | 150 |  |
| 79 | Limit 77 to up=20241127-20260421 |  |  | 89 |

**CINAHL (EBSCO) search conducted on the 22^nd^ of October 2022, the 27^th^ of November 2024 and the 21^st^ of April 2026**

| **Cinahl** | | | | |
| --- | --- | --- | --- | --- |
| **#** | **Search** | **Results**   *October 22, 2022* | **Results**   *November 27, 2024* | **Results** *April 21, 2026* |
| 1 | ( ( (MH "Nurses+") OR (MH "Nursing Practice+") OR (MH "Nursing as a Profession+") OR (MH "Nursing Staff, Hospital") ) OR ( nursing N3 staff OR registered nurse OR nurse practitioners ) ) |  | 527,698 | 574,463 |
| 2 | ( ( (MH "Work Environment+") OR (MH "Health Facility Environment") OR (MH "Job Satisfaction+") OR (MH "Job Experience") OR (MH "Personnel Turnover") OR (MH "Personnel Retention") OR (MH "Nursing Shortage") OR (MH "Workload") OR (MH "Work Engagement") OR (MH "Work-Life Balance") OR (MH "Sick Leave") OR (MH "Nurse-Patient Ratio") OR (MH "Psychological Safety") OR (MH "Occupational Safety") OR (MH "Stress, Occupational+") OR (MH "Occupational-Related Injuries") OR (MH "Workplace Violence") OR (MH "Occupational Health+") OR (MH "Shiftwork") OR (MH "Understaffing") OR (MH "Temporary Employment") OR (MH "Quality of Working Life") OR (MH "Personnel Recruitment") ) OR ( work* N3 environment OR nurse turnover OR work motivarion OR intent-to-leave OR minimum nurse-to-patient ratios ) |  | 229,425 | 252,991 |
| 3 | ( ( (MH "Hospitals+") OR (MH "Mental Health") OR (MH "Ambulatory Care Facilities+") OR (MH "Inpatients") OR (MH "Outpatients") ) OR regional health service ) |  | 334,430 | 367,527 |
| 4 | ( ( ( (MH "Patient Safety") OR (MH "Adverse Health Care Event+") OR (MH "Hospital Mortality") OR (MH "Health Care Errors+") OR (MH "Cross Infection+") OR (MH "Readmission") OR (MH "Violence+") OR (MH "Aggression") OR (MH "Restraint, Physical") OR (MH "Patient Seclusion") OR (MH "Patient Assault") OR (MH "Injuries, Self-Inflicted") OR (MH "Suicide") OR (MH "Outcome Assessment") OR (MH "Quality of Nursing Care") OR (MH "Coercion") ) OR (MH "Nursing Outcomes") OR ( patient harm OR coercion OR self-harm OR nurse sensitive indicators OR nurse sensitive outcome ) OR patient safety |  | 417,803 | 460,673 |
| 5 | S1 AND S2 AND S3 AND S4 | 2,452 | 2,732 | 3,205 |
| 6 | Language: Danish, English, Norwegian, Swedish | 2,256 | 2,505 | 2,927 |
| 7 | DT 2022/10/25-2024/11/27 |  |  |  |
|  | DT 2022/10/25-2026/04/21 |  |  |  |
| 8 | S6 AND S7 |  | 363 | 450 |
