# Supplementary material for: Nursing Work Environments and Patient Safety in Older Adults: A Scoping Review of Their Intricate Relationship, Definitions and Theoretical Frameworks
Source: J Adv Nurs. 2026 Jul 29;82(12):10171–93. doi: 10.1111/jan.70683 (PMC13752672; doi:10.1111/jan.70683)
Supplement: Supplementary file 2 [file JAN-82-10171-s002.docx]

**Elaboration and overview of activities involving knowledge users in the scoping review**

| **Review stage** | **Review task according to JBI guidance** | **Activities on how to include knowledge users in scoping review** | **Overview of activities involving knowledge users in the scoping review process** |
| --- | --- | --- | --- |
| **Before the start of the scoping review process (strategy development)** | Planning for knowledge user engagement | - Reflect on why you want to engage with knowledge users - Identify what type of knowledge user is most appropriate   Consider recruitment and engagement methods | Formed a user involvement group consisting of patients, relatives, RNs, nurse managers and work environment representatives from regional hospitals in Denmark. |
|  | Development of relationship with knowledge users | Develop relationships with knowledge users early to ensure adequate time for relationship building, engagement and mutual expectation setting. Consider developing a Terms of Reference to aid with transparency and accountability | Conducted meetings and workshops with the group to establish rapport, communicate research goals and define roles in contributing to the project. |
|  | Pre-planning and conceptualisation of the review | Enquire with knowledge users about what they feel is important in research. This could be through informal discussions or through research priority setting workshops and surveys in which knowledge users prioritise what areas need further exploration. | Engaged the group through a workshop to discuss the initial concept, review topics and refine focus on nurse recruitment and retention, patient safety and workforce issues. |
| **Protocol development** | Defining objectives and questions;  Developing and aligning inclusion criteria;  Describing the planned approach to the scoping review; | For example, knowledge users can:   - Develop, refine or approve the research questions - Define eligibility criteria - Assist and review in developing search strategy, e.g. identify terms, provide key studies - Provide feedback on data extraction form - Review protocol manuscript | Collaborated with the group to co-create research questions to ensure alignment with real-world challenges and patient safety concerns. Consulted knowledge users to ensure inclusion criteria reflected key issues in nursing practice, particularly nurse shortages and their impact on patient safety.  Provided the user group with a draft of the scoping review protocol, incorporated their feedback on methodologies, inclusion/exclusion. |
| **During the scoping review** | Searching, selecting, extracting and analysing the evidence;  Presenting results;  Summarising evidence in relation to purpose, making conclusions and noting implications of findings; | For example, knowledge users can:   - Take an active role through helping to screen, select and extract articles - Check results to see if they ‘make sense’ - Offer suggestions on the presentation of the results   Help develop or review the implications of the findings to ensure that they are meaningful to the community | Involved the group in refining the data extraction tool and consulted them during the review process to ensure the evidence gathered was applicable to clinical practice.  Presented interim findings to the group for feedback and facilitated discussions on how the results resonated with their experience in nursing practice and patient safety concerns.  Engaged the group in reviewing key findings, discussing implications for practice and shaping conclusions to ensure practical relevance. |
| **After the scoping review** | Dissemination of results; | - Knowledge users were able to participate in the development of evidence summaries, or science communication strategies for social media. They were then able to share the findings from the scoping review through the community   Knowledge users and researchers could advocate for findings to be shared in relevant communities and political organisations to ensure that they are reaching decision-makers. | The group was consulted on how best to communicate findings to healthcare practitioners and stakeholders, providing input on dissemination strategies to maximize impact. |
| ***Note: Adapted from Joanna Briggs Institute and Pollock et al. (2023), and further refined based on the scoping review protocol (Thorsboe et al., 2023)*** | | | |
