# Supplementary material for: Nursing Work Environments and Patient Safety in Older Adults: A Scoping Review of Their Intricate Relationship, Definitions and Theoretical Frameworks
Source: J Adv Nurs. 2026 Jul 29;82(12):10171–93. doi: 10.1111/jan.70683 (PMC13752672; doi:10.1111/jan.70683)
Supplement: Supplementary file 3 [file JAN-82-10171-s003.docx]

| Reference no. | Author, year | Country | Study design | Aim of study | Study focus | Type of unit | Work environment defined | Patient safety defined | Acknowledgement of multimorbidity* | Comorbidity measurement | Theory or framework | Key findings in relation to the relationship between nursing work environments and patient safety |
| --- | --- | --- | --- | --- | --- | --- | --- | --- | --- | --- | --- | --- |
| [1] | Peutere et al., 2024 | 5 | Prospective record linkage | To assess the association between daily nurse understaffing, limited nursing experience, and patient mortality. | 1 | Unknown | I | I | N | CCI | N | Nurse understaffing increased mortality risk, especially in patients with comorbidities when combined with limited nursing experience. |
| [2] | Peutere et al., 2023 | 5 | Longitudinal register-based | To identify low staffing days via RN/RPN work-hour indicators. | 3 | MU; SU | I | I | N | CCI | N | Nurse understaffing and limited experience showed no consistent link to HAIs. Bloodstream infections: Higher risk with less experienced/younger nurses. Surgical site infections: Higher risk with understaffing. |
| [3] | Rochefort et al., 2022 | 4 | Cohort | To estimate associations of RN understaffing, education, experience, and non-RN skill mix with postoperative atrial fibrillation (POAF). | 1 | ICU/CCU; SU | I | I | N | CCI | N | Higher RN understaffing in the first 6 postoperative days was linked to increased POAF. More RN experience reduced POAF risk, while RN education and non-RN mix showed no effect. |
| [4] | Meredith et al., 2024 | 3 | Longitudinal observational | To explore the relationship between nurse staffing  levels and patient outcomes. | 1 | AC; GU; SU | I | I | N | CCI | N | Low RN and NA staffing were associated with higher risks of mortality, readmission, DVT, pneumonia, and pressure ulcers. Each day of understaffing increased risk, with RN understaffing showing stronger effects. Weekend admissions and high patient comorbidity further elevated risks. |
| [5] | Catania et al., 2024 | 7 | Descriptive regression | To examine the association between nurses’ intention to leave, workload, and 30-day in-hospital mortality. | 3 | SU | E | I | N | N/A | Y | Higher nurse workloads and intention to leave are linked to increased patient mortality. BSN education, better work environments, and lower emotional exhaustion improve patient outcomes. |
| [6] | Daus et al., 2024 | 1 | Cross sectional | To examine ethnic differences in 7-, 30-, and 90-day readmission odds, and if these were influenced by the nursing work environment. | 1 | SU | E | I | N | ECS | N | Better nurse work environments are linked to lower readmission rates for older Hispanic surgical patients. |
| [7] | Griffiths et al., 2024 | 3 | Longitudinal observational | To examine the association between nursing team composition and risk of death after acute admission, focusing on staffing levels, RN proportion, seniority, and use of temporary staff. | 1 | ICU/CCU; AC; MU; SU | I | I | N | CCI | Y | Low nurse staffing increases the risk of death, and seniority and experience alone do not offset the impact of staffing shortfalls. |
| [8] | Ambrosi et al., 2017 | 7 | Longitudinal observational | To describe in-hospital mortality and test two hypotheses: H1: higher mortality during weekends; H2: higher mortality with skill mix favouring aides over RNs. | 1 | MU | I | E | Y | N/A | N | Weekend care reductions increased mortality risk, while more RN care hours lowered it. Patients with 12-29 fewer minutes of RN care daily had a higher risk of death, particularly on weekends. |
| [9] | Duffield et al., 2011 | 2 | Longitudinal retrospective, cross-sectional | To examine trends in nursing workload and skill mix over time, and explore their relationships, along with the work environment, to patient outcomes. | 1 | MU; SU; MSU | I | I | N | N/A | Y | Increased RN/CNS staffing reduced decubiti, pneumonia, and sepsis rates. Unsafe environments and resource shortages worsened care and increased overtime. Higher turnover and care hours led to more falls. Familiar staff reduced errors. |
| [10] | Fagerström et al., 2018 | 5 | Observational | To examine whether daily workload per nurse correlates with patient safety incidents and mortality. | 1 | MU; SU; PU; RU; G; M; N; O; ON; L; OT | I | E | N | N/A | N | Exceeding optimal OPC/nurse levels increased patient safety incidents. Staying below optimal levels decreased safety incidents. |
| [11] | He et al., 2016 | 1 | Longitudinal observational | To examine the association between nurse staffing and patient outcomes. | 1 | ICU/CCU; SDU; MU; SU; MSU; RU | I | I | N | N/A | N | Total HPPD was inversely related to all outcomes, while a higher RN skill mix was linked to increased fall and pressure ulcer rates. Falls and ulcers were more likely when HPPD was lower and RN skill mix higher. |
| [12] | Lasater & McHugh, 2016 | 1 | Cross sectional | To examine the effect of nurse staffing and work environment on 10- and 30-day unplanned readmissions after elective hip/knee replacement. | 1 | SU | E | E | N | N/A | N | Patients in optimal work environments had lower odds of 30-day readmission after major joint replacement. |
| [13] | Staggs et al., 2017 | 1 | Longitudinal observational | To examine whether nurse staffing is associated with restraint. | 1 | MU; SU; MSU | I | E | y |  | N | Lower skill mix increased restraint use. An increase in skill mix reduced restraint use. |
| [14] | Kutney-Lee et al., 2017 | 1 | Longitudinal observational, retrospective panel | To examine whether increases in BSN-prepared nurses were associated with mortality and FTR, controlling for staffing, skill mix, and RN experience. | 1 | SU | I | I | N | ECS | N | Higher proportions of BSN-educated nurses are linked to lower surgical mortality. |
| [15] | Kutney-Lee et al., 2021 | 1 | Cross sectional | To examine associations between EHR usability, nurse job outcomes, and patient outcomes. | N/A | ICU/CCU; MSU | I | I | N | ECS | N | Nurses in hospitals with poor EHR usability had higher odds of burnout, job dissatisfaction, and intention to leave. Surgical patients faced higher mortality and 30-day readmission. Comprehensive EHR adoption increased burnout. |
| [16] | Aiken et al., 2014 | 15 | Observational | To assess whether patient-to-nurse ratios and nurse education levels are associated with hospital mortality. | 1 | SU | I | I | N | CCI | N | Significant reduction in mortality associated with improved nurse education levels. |
| [17] | Ball et al., 2018 | 15 | Observational | To examine whether missed nursing care mediates the link between nurse staffing and mortality. | 1 | SU | E | E | N | CCI | N | Missed nursing care raised post-surgical mortality. Each additional patient per nurse increased 30-day mortality, while a rise in missed care raised it. An increase in BSN nurses reduced this risk. |
| [18] | Salvador et al., 2021 | 1 | Longitudinal | To examine the effect of unit-level functional flexibility on pressure ulcers, and whether coworker support and workload moderate the relationship. | 1 | ICU/CCU; SU | I | I | N | N/A | Y | Higher use of functionally flexible nurses increases pressure ulcers, but strong coworker support reduces this effect. |
| [19] | Dietermann et al., 2021 | 8 | Cross sectional | To provide evidence on how nurse staffing levels impact seven nursing-sensitive patient outcomes. | 1 | MU; SU | I | I | N | ECS | N | Nurse staffing levels are associated with several nursing-sensitive patient outcomes, with effects varying by unit type and severity. Strongest associations for pneumonia, respiratory failure, and pressure ulcers. |
| [20] | Needleman et al., 2011 | 1 | Retrospective observational | To examine associations between mortality and exposure to RN understaffed shifts and high patient turnover. | 1 | ICU/CCU; SDU; MU, SU | I | I | N | ECS | N | Increased mortality was significantly linked to below-target RN staffing and high patient turnover. |
| [21] | Aiken et al., 2011 | 1 | Comparative | To determine how nurse staffing, education, and work environment relate to patient outcomes. | 1 | SU | E | I | N | ECS | N | Improved staffing reduced surgical mortality only in hospitals with good work environments. BSN education benefits outcomes across all hospitals but staffing investments yield the greatest value when paired with supportive environments. |
| [22] | Bettencourt et al., 2020 | 1 | Retrospective observational | To analyze the relationship between nurse staffing, work environment, and mortality. | 1 | Unknown | E | I | N | ECS | N | Each additional patient in a nurse’s workload is linked to an increase in mortality odds. Improving the work environment is associated with a reduction in death odds. |
| [23] | Ma et al., 2015 | 1 | Retrospective, cross sectional | To examine how the nurse work environment, staffing, and education relate to 30-day readmissions. | 1 | SU | I | I | N | ECS | N | Each SD increase in PES-NWI score correlates with a decrease in 30-day readmission odds. Improvements in care quality and support lowered readmission odds. Each additional patient per nurse increased readmission odds. Better work environments reduced readmission risk regardless of staffing levels. |
| [24] | Schlak et al., 2021 | 1 | Cross sectional | To explore associations between nurse burnout and mortality, FTR, and LOS, considering the work environment. | 1 | SU | E | I | N | ECS | N | Higher nurse burnout scores were associated with increased odds of mortality, FTR, and longer LOS. Positive work environments mitigated these effects. Hospitals with Magnet status showed reduced associations between nurse burnout, mortality, and FTR. |
| [25] | Twigg et al., 2012 | 2 | Retrospective analysis | To examine the association between skill mix and 14 nursing-sensitive outcomes after implementing NHPPD. | 1 | MU; SU | I | I | N | N/A | N | Increased RN hours led to significant reductions in eight nursing-sensitive outcomes, including pressure ulcers and pneumonia, particularly in hospitals with the lowest skill mix. Higher skill mix was linked to decreased rates of various nursing-sensitive outcomes. |
| [26] | Dubois et al., 2013 | 4 | Cross sectional | To examine associations between four nursing care organization models and patient safety outcomes, based on staffing, skill mix, scope of practice, work environment, and unit innovation. | 3 | MU | E | E | N | CCI | N | The risk of experiencing adverse events was significantly lower in professional nursing models compared to functional models. The professional model demonstrated better performance due to richer skill mix, higher staffing intensity, and a supportive practice environment. |
| [27] | Wieczorek-Wojcik et al., 2020 | 9 | Retrospective observational longitudinal | To assess the impact of daily NHPPD on missed care and in-hospital mortality. | N/A | MU; SU | I | I | N | N/A | N | An additional hour of NHPPD in medical departments is associated with a decrease in mortality rates per 1,000 patient days and a lower chance of unplanned deaths. Increasing nursing care correlates with fewer deaths in both SU and MU; an increase in the nursing rate results in a reduction of in-hospital mortality. |
| [28] | Xue et al., 2012 | 1 | Retrospective multilevel longitudinal | To examine the relationship between the use of specialized RNs and patient outcomes. | N/A | ICU/CCU; SDU; MU; SU | E | I | N | CCI | N | The use of specialized RNs varied across units, ranging from 0% to 30.4% of total RN hours, with an average of 9.8% in non-ICUs and 6.4% in ICUs. All observed effects of specialized RN use on patient outcomes were nonsignificant. |
| [29] | Park et al., 2012 | 1 | Descriptive correlational | To examine how the relationship between nurse staffing and patient outcomes varied with the level of patient turnover. | 1 | ICU/CCU; MU; SU; MSU | E | E | N | N/A | Y | Higher RN staffing is associated with lower FTR rates, but the positive effect diminishes as patient turnover increases in non-ICUs. An increase in turnover reduces the staffing benefits on FTR. Not significant in ICUs. |
| [30] | Aiken et al., 2013 | 1 | Cross sectional | To determine the association between the use of 9088 supplemental RNs to staff hospitals and patient mortality and FTR. | 1 | SU | I | I | N | ECS | N | Supplemental RNs contribute positively to patient care and workforce diversity. Hospital leaders should assess whether poor work environments hinder their ability to attract and retain qualified permanent nurses, which may negatively impact patient outcomes. |
| [31] | Park et al., 2014 | 1 | Longitudinal | To examine the association between nurse turnover and the patient outcome of pressure ulcers. | 1 | SDU, MU; SU, MSU | I | I | N | N/A | Y | An increase in RN turnover raises UAPUs. Higher turnover correlates with lower RN staffing levels, while increased staffing reduces UAPU rates. Concurrent turnover doesn't affect UAPUs, lagged turnover does. Higher turnover leads to lower staffing, but staffing levels do not significantly influence turnover rates. |
| [32] | Park et al., 2015 | 1 | Descriptive-correlational | To compare different nursing hours measures in predicting patient outcomes and identify the most accurate staffing metric. | 1 | ICU/CCU | I | I | N | N/A | N | Models using staffing measures based on productive hours from direct-care providers performed best. Separating nonproductive and indirect-care hours from productive direct-care hours has minimal impact on the relationship between staffing and patient outcomes. |
| [33] | Schreuders et al., 2015 | 2 | Retrospective longitudinal | To examine how patient exposure to nurse staffing relates to eight complications. | 1 | ICU/CCU; SDU; MU; SU | I | E | N | CCI | N | Improved staffing reduced surgical wounds and UTIs but increased odds of pressure ulcers, pneumonia, shock, myocardial infarction, and 30-day mortality. FTR decreased with better staffing, but odds increased on high-intensity units. |
| [34] | Giuliano et al., 2016 | 1 | Observational | To examine the relationship between nurse staffing and 30-day excess readmissions for heart failure patients. | 1 | Unknown | I | E | N | N/A | N | The low nurse staffing index group had a higher mean excess readmission ratio compared to the high nurse staffing index group. Hospitals with lower nurse staffing had a significantly higher excess readmission rate for heart failure patients than those with higher staffing levels. |
| [35] | Li et al., 2016 | 1 | Cohort | To determine the effects of RN staffing on HACs in cardiac surgery patients. | 1 | SU | I | E | N | ECS | Y | Higher RN staffing was associated with a reduction in HACs, mortality, and in mean LOS. HAC-associated costs were higher in high-staffing hospitals. |
| [36] | Twigg et al., 2016 | 2 | Quasi-experimental pretest-posttest control group | To assess the impact of adding assistants in nursing to acute care hospital ward nurse staffing on adverse patient outcomes. | 1 | MU; SU; RU | I | I | N | CCI | Y | Significant increases in adverse outcomes on assistant in nursing wards for FTR, UTIs, and falls with injury, while mortality decreased. On non-assistant wards, pneumonia decreased, and falls with injury increased. Time spent on assistant wards predicted UTIs and pneumonia, with increase in time linked to a rise in UTI odds and a rise in pneumonia odds. |
| [37] | Haegdorens et al., 2019 | 10 | Observational | To explore the relationship between nurse staffing and patient mortality, accounting for nurse education, team composition, age, and comorbidity. | 1 | MU; SU | I | I | N | CCI | N | A negative association was found between NHPPD and the composite mortality rate and between the proportion of bachelor hours and the composite mortality rate. Wards with higher NHPPD had lower proportions of unexpected death, CPR, and death after CPR. There was a negative relationship between NHPPD and the combined mortality rate from CPR, unplanned ICU admissions, and unexpected deaths. |
| [38] | Rochefort et al., 2020 | 4 | Longitudinal cohort | To estimate the associations of RN understaffing, education, experience, and non-RN skill mix with hospital mortality. | 1 | ICU/CCU; MU; MSU | I | I | N | CCI | N | An increase in understaffed shifts was associated with an increase in mortality, while an increase in hours worked by BSN-prepared RNs correlated with a reduction in mortality. Patients cared for by a higher proportion of BSN-prepared RNs had a significant survival advantage, independent of RN understaffing. |
| [39] | Fogg et al., 2021 | 3 | Cohort | To explore associations between RN/NA staffing levels, mortality, and readmissions. | 3 | MU; SU | I | I | Y | CCI | N | Increasing RN hours per day was associated with reduced mortality and greater benefits for patients with CI. Additional NA hours increased mortality risk in patients without CI. Higher RN staffing improved outcomes, particularly for those with CI, while higher NA staffing reduced mortality for patients with CI but increased it for those without. |
| [40] | Falk, 2022 | 11 | Observational | To investigate if differences in patient characteristics and nurse–patient ratio have an impact on the quality of care. | 1 | MU; SU | I | I | N | N/A | N | No difference in mortality or LOS between the units; however, the unit with a 1:1 nurse-patient ratio experienced more complications, including unplanned re-intubations and longer duration on mechanical ventilation. |
| [41] | Roberts et al., 2022 | 2 | Retrospective observational | To examine associations between critical incidents and nursing shift rosters in the ED. | N/A | EM | I | E | N | N/A | Y | The analysis of early, late, and night shifts over 48- and 96-hour periods found no significant relationship between CI and nursing shift patterns. However, the LOS in the ED was significantly higher for patients with CI. |
| [42] | Frith et al., 2010 | 1 | Cross sectional | To examine the predictive relationships between nurse staffing and patient outcomes in hospitals. | 1 | MSU | E | E | N | N/A | N | Higher RN and LPN HPPD, and a higher percentage of RNs in the skill mix, were associated with fewer adverse events and shorter LOS. HAIs were the most common, followed by pressure ulcers and CAUTIs. The influence of RN hours on LOS was stronger than that of LPN hours. |
| [43] | Trinkof et al., 2011 | 1 | Cross sectional | To determine if, in hospitals where nurses report more adverse work schedules, there would be increased patient mortality, controlling for staffing. | 1 | Unknown | I | I | N | N/A | N | Higher mortality from AAA, congestive heart failure, and acute myocardial infarction was linked to long nurse hours, insufficient time off, and working while sick. High-mortality hospitals had lower licensed staffing for pneumonia, congestive heart failure, and stroke, and a lower skill mix for acute myocardial infarction and craniotomy. |
| [44] | Hart et al., 2011 | 1 | Retrospective, descriptive and correlational | To evaluate the relationships between staffing indicators and patient outcomes at the hospital. | 1 | ICU/CCU; MSU; T | I | E | N | N/A | Y | No relationship between agency RN hours and patient outcomes. A higher percentage of agency RN hours was linked to increased HAPUs in medical-surgical units. Significant relationship between total nursing care HPPD and RN HPPD with HAPUs, where higher nursing care hours were associated with fewer pressure ulcers. Higher LPN HPPD correlated with reduced restraint use. |
| [45] | Kendall-Gallagher et al., 2011 | 1 |  | To determine if staff nurses with specialty certification is associated with risk-adjusted inpatient 30-day mortality and FTR. | 1 | SU | I | I | N | ECS | N | A higher proportion of BSN and certified BSN staff nurses in hospitals was associated with lower mortality and improved FTR rates. |
| [46] | Staggs et al., 2012 | 1 | Longitudinal observational | To explore characteristics, including nurse staffing, as predictors of unassisted fall rates. | N/A | ICU/CCU; SDU; MU; SU; MSU; RU | I | E | N | N/A | N | The fall rate showed a positive relationship with total nurse staffing at lower levels, while it decreased at staffing levels around and above the median. A higher RN skill mix and greater average RN tenure on the unit were associated with lower fall rates. |
| [47] | Boltz et al., 2013 | 1 | Descriptive | To examine the relationship between nurse certification and unit-level, nursing-sensitive quality indicators. | 3 | MU; MSU | I | E | N | N/A | N | Nurse certification may impact nursing-sensitive outcomes. The results indicated a negative relationship between nurse certification and fall rates, but no correlation with injurious falls, UAPUs, or restraint prevalence. |
| [48] | JiSunChoi et al., 2014 | 1 | Descriptive, correlational | To examine correlations among six nurse staffing measures and their relation to UAPUs. | N/A | ICU/CCU; SDU; MU; SU; MSU | I | I | N | N/A | N | Two versions of HPPD and RN skill mix were correlated with the number of patients assigned to RNs. These measures showed weaker correlations with RNs' perceived staffing adequacy. Among the six staffing variables examined, only RN-perceived staffing adequacy and RN skill mix were associated with the odds of UAPUs. |
| [49] | Cottney et al., 2015 | 3 | Observational | To identify medication administration error types, consequences, and risk factors. | 2 | MHCU | I | E | N | N/A | N | Errors during medication rounds were incorrect dose omission, incorrect dose, incorrect form, and incorrect time. Risk factors included nurse interruptions during medication rounds, a higher number of "when required" doses, and an increased patient load. |
| [50] | Staggs et al., 2016 | 1 | Each unit its own control | To examine if variation in staffing levels affects odds of injurious assault. | 2 | GC | I | I | N | N/A | N | No relationship between RN or non-RN staffing levels and assault odds. Monthly staffing deviations were not linked to assault rates. |
| [51] | Rao et al., 2017 | 1 | Cross sectional | To examine the relationship between nurse autonomy and 30-day mortality and FTR. | 1 | SU | E | I | N | ECS | N | Increased nurse autonomy at the hospital level is linked to reduced odds of 30-day mortality and FTR for surgical patients. |
| [52] | Olds et al., 2017 | 1 | Cross sectional | To determine if safety climate and the nurse work environment contribute to patient outcomes. | 1 | SU | E | E | N | ECS | Y | Better work environment scores were linked to lower mortality odds. |
| [53] | Harrison et al., 2019 | 1 | Cross sectional | To assess how nurse education level and staffing relate to survival with good cerebral outcome after cardiac arrest. | 1 | ICU/CCU; MSU | I | I | N | N/A | N | Hospitals with a higher percentage of BSN-nurses and lower patient-to-nurse ratios had improved odds of patients surviving to discharge with good cerebral performance after in-hospital cardiac arrest (IHCA). Higher patient-to-nurse ratios negatively impacted survival chances. |
| [54] | Needleman et al., 2020 | 1 | Survival analysis | To examine the association of mortality with patients’ exposure to shifts with low RN staffing, low nursing support staffing, and high patient turnover. | 1 | ICU/CCU; SDU; MU; MSU | I | I | N | CCI | N | Shifts with low RN staffing or low nursing support were associated with increased hazard ratios for adverse outcomes. The combination of low staffing levels showed significant risks, however, no relationship between high patient turnover and mortality. |
| [55] | Dall’Ora et al., 2020 | 3 | Retrospective, longitudinal, observational | To examine the association between the levels of temporary staffing to the risk of death. | 1 | MU; SU | I | I | N | CCI | N | High levels of temporary RN staffing raised the risk of death, while excessive temporary NA staffing also contributed to higher mortality risk. Higher amounts of temporary RN staffing are associated with increased patient risk. |
| [56] | Flanagan et al., 2020 | 1 | Cross sectional | To examine hospital characteristics and staffing levels, and how they may affect HACs associated with rates of sepsis. | 1 | ICU/CCU; SDU; MSU | I | I | N | N/A | N | Higher patient-to-nurse ratios correlated with increased sepsis rates, with the risk rising as nurses cared for more patients. Increased staffing and intensivist hours were linked to lower sepsis rates. |
| [57] | Lake et al., 2010 | 1 | Cross sectional | To examine the relationships among nurse staffing, RN composition, hospitals’ Magnet status, and patient falls. | N/A | ICU/CCU; SDU; MU; SU; MSU; RU | I | I | N | N/A | Y | Magnet recognition and increased RN hours were linked to fewer patient falls, while more LPN and NA hours were associated with higher fall rates. BSN nurse proportions and agency hours showed no effect. |
| [58] | Bae et al., 2010 | 1 | Nonexperimental, longitudinal causal modeling | To investigate the association between the use of temporary nurses and patient safety outcomes. | 1 | MU; SU; MSU | E | E | N | N/A | N | Nurses in units with high levels of external temporary RN staffing reported more back injuries and higher rates of patient falls. Units with moderate levels of external temporary RN hours experienced fewer medication errors. |
| [59] | Esparza et al., 2012 | 1 |  | To examine the impact of nurse staffing and RN skill mix on patient outcomes. | 1 | MSU | I | I | N | N/A | N | Increased total NHPPD and a higher proportion of RN hours were linked to lower odds of UTIs and shorter LOS. As NHPPD rose, the odds of developing a UTI decreased, and a greater proportion of RNs in the nursing staff reduced both UTI odds and LOS. |
| [60] | WiltseNicely et al., 2013 | 1 | Cross sectional | To determine if lower mortality rates for patients undergoing abdominal aortic aneurysm (AAA) repair is explained by better nursing. | 3 | SU | I | I | N | ECS | N | Favorable nursing practice environments and higher volumes of AAA repairs correlate with lower mortality rates and fewer FTR. In poorly staffed hospitals, increased volume had little impact on mortality. Patients in well-staffed hospitals with high volumes of AAA repairs had lower mortality rates compared to those in poorly staffed facilities. |
| [61] | Choi et al., 2013 | 1 | Cross sectional | To examine the relationship between RN workgroup job satisfaction and HAPUs. | 1 | AC; SDU; MU; SU; MSU; RU | E | E | N | N/A | Y | Higher RN workgroup job satisfaction is associated with a lower likelihood of HAPUs. For MU and RU the odds of HAPU occurrence decrease with increased RN job satisfaction. No relationship in SDU, SU, or MSU. RN unit tenure was found to correlate positively with lower HAPU rates, with each year of increased tenure associated with a decrease in HAPU occurrence. |
| [62] | Talsma et al., 2014 | 1 | Cohort, multisite | To investigate variation in ICU/general care unit staffing levels and its association with FTR mortality. | 1 | ICU/CCU; GU | I | E | N | N/A | Y | Higher RN HPPD levels suggested increased odds of FTR mortality. In ICUs, no associations were found between nurse staffing variables and FTR mortality. |
| [63] | Kelly et al., 2014 | 1 | Cross sectional | To examine whether variation in nurse staffing, environment, education, and experience is associated with mortality. | 1 | ICU/CCU | E | I | N | ECS | N | Better work environments correlated with an 11% reduction in mortality risk. For each 10% increase in the proportion of ICU nurses holding a BSN, there was a 2% decrease in the odds of 30-day mortality. Critical care staffing levels and nurse experience did not impact mortality outcomes once other nurse characteristics were considered. |
| [64] | Ma et al., 2015 | 1 | Cross sectional | To examine how unit work environment and Magnet status relate to HAPUs. | 1 | MU; SU; MSU | E | E | N | N/A | N | Magnet hospital units demonstrated a 21% lower likelihood of having HAPUs compared to non-Magnet units. An increase in PES-NWI score was associated with a 29% reduction in HAPU odds. Magnet hospitals reported significantly lower HAPU rates, better overall work environments, and a higher proportion of nursing hours provided by RNs compared to non-Magnet units. |
| [65] | McHugh et al., 2016 | 1 | Cross sectional | To determine the association between nurse staffing, nurse work environments, and IHCA survival. | 1; 3 | ICU/CCU; MSU | E | I | N | N/A | N | Each additional patient per nurse was linked to a 5% decrease in the likelihood of surviving an IHCA to discharge. Patients in hospitals with poor work environments had a 16% lower chance of survival following an IHCA compared to those in hospitals with better work environments. |
| [66] | Drews et al., 2019 | 1 | Observational | To assess the relationship between interruption frequency and common patient hazards. | 1 | ICU/CCU | I | I | N | N/A | N | Tasks interrupted by device alarms were linked to higher rates of care delays and safety hazards, while interruptions from humans did not increase these rates. |
| [67] | Shang et al., 2019 | 1 | Cross sectional | To examine whether HAIs and nurse staffing are associated. | 1 | ICU/CCU; MU; SU; MSU | I | I | N | CCI | N | HAIs were associated with RN and nursing support understaffing. Risks increased by 15% with RN understaffing and 11% with nursing support understaffing on both shifts two days before infection. No risk when only one shift was understaffed. |
| [68] | Margadant et al., 2020 | 12 | Retrospective analysis | To examine the association between nursing workload and in-hospital mortality. | 1 | ICU/CCU | I | I | N | N/A | N | A Nursing Activities Score per nurse ratio greater than 41 was linked to higher in-hospital mortality. After adjusting for case mix, ratios above 61 remained significantly associated with increased mortality risk. The patient-to-nurse ratio was not related to in-hospital mortality. |
| [69] | Lasater et al., 2021 | 1 | Retrospective matched-cohort | To compare outcomes and costs between hospitals with better vs. worse nursing resources and assess patient mortality risk. | 1 | SU; GU; O | I | I | N | N/A | N | Patients in hospitals with better nursing resources experienced lower 30-day mortality, FTR, readmissions, and shorter LOS compared to those in hospitals with poorer resources. Costs were marginally higher in better-resourced hospitals, but the associated benefits in patient outcomes were substantial. |
| [70] | Juvé-Udina et al., 2020 | 6 | Observational, descriptive | To compare patient acuity, RN staffing and workforce measures, missed nursing care, and patient outcomes. | 3 | SDU; MU; SU | I | I | Y | N/A | N | General wards experience higher rates of mortality, skin injuries, and family compassion fatigue. SDUs report higher occurrences of line-associated phlebitis, delirium, and falls. Structural understaffing results in an average of 21% missed nursing care. |
| [71] | Lasater et al., 2021 | 1 | Matched cohort | To determine whether there are differential clinical outcomes, costs, and value at hospitals characterized by better or worse nursing resources. | 1 | ICU/CCU; GU | I | I | N | N/A | N | Patients in hospitals with better nursing resources experienced significantly lower 30-day mortality and fewer readmissions, with no significant difference in costs. Hospitals with superior nursing resources achieved better patient outcomes without increased costs. |
| [72] | Porcel-Gálvez et al., 2020 | 6 | Prospective observational cohort | To identify which patient and hospital factors are related to hospital mortality. | 1 | MU; SU | I | E | Y | N/A | N | Higher nursing care dependency was linked to lower odds of adverse outcomes, while lower staffing levels increased odds. Risk factors included older age, male gender, admission to medical units or secondary-care hospitals, and winter admissions. |
| [73] | Dzikowicz et al., 2020 | 1 | Prospective cohort | To compare patient complications, patient and staff satisfaction between all-RN and mixed-skill units. | 1 | MU | I | I | N | N/A | N | The all-RN unit had lower rates of patient falls and CLABSI than the control unit, with no reported CAUTIs. Reductions in fall-related injuries and pressure injuries were not statistically significant. The unit saw decreased 30-day readmissions. |
| [74] | Lasater et al., 2021 | 1 | Cross sectional | To evaluate whether patient-to-nurse staffing ratios are associated with clinical outcomes for patients admitted with sepsis. | 1 | MSU | I | I | N | ECS | N | Each additional patient per nurse increases the odds of in-hospital and 60-day mortality, readmissions, and LOS. Although adherence to sepsis treatment guidelines reduces mortality and LOS, nurse staffing levels have a stronger overall impact. |
| [75] | Fasoi et al., 2020 | 13 | Cross sectional | To measure nursing workload (NW), correlate it with mortality, and determine required nurse-to-patient ratios. | 1 | ICU/CCU | I | I | N | N/A | N | NW was not correlated with patient age or gender but showed a positive correlation with the workload of both survivors and non-survivors, as well as patients with long and short ICU stays. |
| [76] | Scheier et al., 2021 | 14 | Prospective | To investigate the impact of employee turnover on CLABSI incidence. | 1 | ICU/CCU | I | I | N | N/A | N | A correlation was found between CLABSI incidence rates and LPNs, as well as RNs. Physician turnover showed no correlation with CLABSI incidence. |
| [77] | Kumar et al., 2022 | 1 | Cohort | To determine relationships between nursing resources and mortality in patients with and without depression. | 2; 1 | SU | E | E | Y | ECS | N | Each additional patient per nurse was associated with a 4% increase in mortality odds for patients with depression. A 10% increase in the proportion of BSN nurses correlated with a 4% decrease in mortality odds, and a one SD improvement in the work environment led to a 5% decrease in mortality odds. |
| [78] | Moronski et al., 2025 | 1 | Cross‑sectional correlational study | To examine associations between nurse staffing levels and 30‑ and 60‑day readmissions among adults with intellectual and developmental disability (IDD) | 3 | AC; GU | I | I | Y | ECS | N | Higher nurse workload was associated with worse patient safety outcomes: each additional patient per nurse increased odds of 30‑day readmission by 7% and 60‑day readmission by 9% among patients with IDD. |
| [79] | Fernández-Sánchez et al., 2025 | 6 | Descriptive cross‑sectional observational study | To examine the relationship between nurse–patient ratios and adverse events in adult conventional inpatient units | 1 | MU; SU; GU | I | I | I | N/A | N | Higher nurse-patient ratios were associated with increased adverse events, particularly hospital‑acquired pressure injuries (HAPI). Each 1‑point increase in 24‑h ratio increased HAPI risk. Associations were weaker or non‑significant in surgical units. |
| [80] | Lasater et al., 2025 | 4 | Cross‑sectional observational study | To evaluate nurse staffing, nurse job outcomes, patient safety, care quality, mortality and readmissions in BC hospitals prior to implementation of minimum nurse‑to‑patient ratios | 3 | GU; AC | I | I | Y | ECS | N | Higher patient‑to‑nurse ratios were associated with worse patient safety outcomes, including higher risk‑adjusted mortality, higher 30‑day readmission rates, more nurse‑reported adverse events, poorer patient safety culture, and more missed nursing care. |
| [81] | Iroegbu et al., 2026 | 1 | Cross‑sectional observational study | To examine associations between nurse staffing, nurse education, and 30‑day mortality among patients hospitalized with acute kidney injury (AKI) | 1 | MU; MSU | I | I | I | ECS | N | Each additional patient per RN increased odds of 30‑day mortality, while higher proportions of BSN‑prepared nurses were associated with lower 30‑day mortality among AKI patients. |
| [82] | Tack et al., 2025 | 10 | Prospective cohort study | To assess the association between nursing workload at ICU discharge and risk of ICU readmission | 3 | ICU | E | I | I | CCI | N | Higher nursing workload at ICU discharge was independently associated with increased risk of ICU readmission. |
| [83] | Moscelli et al., 2024 | 3 | Retrospective longitudinal panel study | To examine associations between nurse and senior doctor turnover and patient outcomes in NHS acute trusts | 3 | GU; AC | I | I | Y | CCI | N | Higher nurse and doctor turnover were associated with increased 30‑day mortality, particularly for emergency admissions. |
| [84] | Muir et al., 2026 | 1 | Cross‑sectional observational study using linked nurse survey and administrative patient data | To examine associations between emergency department and medical‑surgical nurse staffing and patient departures against medical advice (AMA) | 3 | EM; MSU | I | I | I | ECS | N | Higher nurse workloads in both emergency departments and medical‑surgical units were associated with higher odds of patients leaving against medical advice. |

**Note:**

***Co-existing physical and mental illness**

**Comorbidity measurement:** ECS = Elixhauser Comorbidity Score, CCI = Charlson Comorbidity Index

**Country:** 1 = United States of America, 2 = Australia, 3 = The United Kingdom, 4 = Canada, 5 = Finland, 6 = Spain, 7 = Italy, 8 = Germany, 9 = Poland, 10 = Belgium, 11 = Sweden, 12 = The Netherlands, 13 = Greece, 14 = Switzerland, 15 = RN4CAST (multiple countries)

**Work environment definition:** I = Implicit defined, E = Explicit defined

**Patient safety definition:** I = Implicit defined, E = Explicit defined

**Study focus:** 1 = Physical health, 2 = Mental health, 3 = Multi diagnosis

**Type of unit:** SU = Surgical unit, MU = Medical unit, ICU/CCU = Intensive Care Unit/Critical Care Unit, MSU = Medical-surgical Unit, SDU = Step-down Unit, RU = Rehabilitation Unit, PU = Pediatric Unit, G = Gynaecology, M = Maternity, N = Neurology, O = Orthopedic, ON = Oncology, L = Lung, OT = Otology, T = Telemetry, MHCU = Mental Health Care Unit, GC = Geriatric Care, AC = Acute Care, GU = General Unit, EM = Emergency Medicine

N/A = Not applicable

**References**

**[1]** Peutere, L., Pentti, J., Ropponen, A., Kivimäki, M., Härmä, M., Krutova, O., Ervasti, J., Koskinen, A., Virtanen, M., 2024. Association of nurse understaffing and limited nursing work experience with in-hospital mortality among patients: A longitudinal register-based study. International Journal of Nursing Studies 150, 104628. <https://doi.org/10.1016/j.ijnurstu.2023.104628>

**[2]** Peutere, L., Terho, K., Pentti, J., Ropponen, A., Kivimäki, M., Härmä, M., Krutova, O., Ervasti, J., Koskinen, A., Virtanen, M., 2023. Nurse Staffing Level, Length of Work Experience, and Risk of Health Care-Associated Infections Among Hospital Patients: A Prospective Record Linkage Study. Med Care 61, 279–287. <https://doi.org/10.1097/MLR.0000000000001843>
**[3]** Rochefort, C.M., Bourgon Labelle, J., Farand, P., 2022. Nurse Staffing Practices and Postoperative Atrial Fibrillation Among Cardiac Surgery Patients: A Multisite Cohort Study. CJC Open 4, 37–46. <https://doi.org/10.1016/j.cjco.2021.08.013>
**[4]** Meredith, P., Turner, L., Saville, C., Griffiths, P., 2024. Nurse understaffing associated with adverse outcomes for surgical admissions. BRITISH JOURNAL OF SURGERY 111. <https://doi.org/10.1093/bjs/znae215>
**[5]** Catania, G., Zanini, M., Cremona, M.A., Landa, P., Musio, M.E., Watson, R., Aleo, G., Aiken, L.H., Sasso, L., Bagnasco, A., 2024. Nurses’ intention to leave, nurse workload and in-hospital patient mortality in Italy: A descriptive and regression study. Health Policy 143, 105032. <https://doi.org/10.1016/j.healthpol.2024.105032>

**[6]** Daus, M., McHugh, M.D., Kutney-Lee, A., Brooks Carthon, M.J., 2024. Effect of the Nurse Work Environment on Older Hispanic Surgical Patient Readmissions. Nursing Research 73, E1. <https://doi.org/10.1097/NNR.0000000000000698>

**[7]** Griffiths, P., Saville, C., Ball, J., Culliford, D., Jones, J., Lambert, F., Meredith, P., Rubbo, B., Turner, L., Dall’ora, C., Workforce Health Outcomes Study Group, Barraclough, A., Bojke, C., Bramley, L., Bull, G., Carmona, L., Charlwood, A., Drennan, J., Fillingham, J., Flynn, L., Hyett, F., Redfern, O., Rix, L., Scmidt, P., Shields, D., Stiles, C., Swinson, K., Taylor, R., Tape, N., 2024. Nursing Team Composition and Mortality Following Acute Hospital Admission. JAMA Netw Open 7, e2428769. <https://doi.org/10.1001/jamanetworkopen.2024.28769>

**[8]** Ambrosi, E., De Togni, S., Guarnier, A., Barelli, P., Zambiasi, P., Allegrini, E., Bazoli, L., Casson, P., Marin, M., Padovan, M., Picogna, M., Taddia, P., Salmaso, D., Chiari, P., Frison, T., Marognolli, O., Canzan, F., Saiani, L., Palese, A., 2017. In-hospital elderly mortality and associated factors in 12 Italian acute medical units: findings from an exploratory longitudinal study. Aging clinical and experimental research 29, 517–527. <https://doi.org/10.1007/S40520-016-0576-8>

**[9]** Duffield, C., Diers, D., O’Brien-Pallas, L., Aisbett, C., Roche, M., King, M., Aisbett, K., 2011. Nursing staffing, nursing workload, the work environment and patient outcomes. Applied Nursing Research 24, 244–255. <https://doi.org/10.1016/j.apnr.2009.12.004>

**[10]** Fagerström, L., Kinnunen, M., Saarela, J., 2018. Nursing workload, patient safety incidents and mortality: an observational study from Finland. BMJ open 8. <https://doi.org/10.1136/BMJOPEN-2017-016367>

**[11]** He, J., Staggs, V.S., Bergquist-Beringer, S., Dunton, N., 2016. Nurse staffing and patient outcomes: a longitudinal study on trend and seasonality. BMC nursing 15. <https://doi.org/10.1186/S12912-016-0181-3>

**[12]** Lasater, K.B., Mchugh, M.D., 2016. Nurse staffing and the work environment linked to readmissions among older adults following elective total hip and knee replacement. International journal for quality in health care : journal of the International Society for Quality in Health Care 28, 253–258. <https://doi.org/10.1093/INTQHC/MZW007>

**[13]** Staggs, V.S., Olds, D.M., Cramer, E., Shorr, R.I., 2017. Nursing Skill Mix, Nurse Staffing Level, and Physical Restraint Use in US Hospitals: a Longitudinal Study. Journal of general internal medicine 32, 35–41. <https://doi.org/10.1007/S11606-016-3830-Z>
**[14]** Kutney-Lee, A., Sloane, D.M., Aiken, L.H., 2017. An Increase In The Number Of Nurses With Baccalaureate Degrees Is Linked To Lower Rates Of Postsurgery Mortality. <https://doi.org/10.1377/hlthaff.2012.0504> 32, 579–586. <https://doi.org/10.1377/HLTHAFF.2012.0504>
**[15]** Kutney-Lee, A., Carthon, M., Sloane, D., Bowles, K., McHugh, M., Aiken, L., 2021. Electronic Health Record Usability Associations With Nurse and Patient Outcomes in Hospitals. MEDICAL CARE 59, 625–631. <https://doi.org/10.1097/MLR.0000000000001536>
**[16]** Aiken, L., Sloane, D., Bruyneel, L., Van den Heede, K., Griffiths, P., Busse, R., Diomidous, M., Kinnunen, J., Kozka, M., Lesaffre, E., McHugh, M., Moreno-Casbas, M., Rafferty, A., Schwendimann, R., Scott, P., Tishelman, C., van Achterberg, T., Sermeus, W., RN4CAST Consortium, 2014. Nurse staffing and education and hospital mortality in nine European countries: a retrospective observational study. LANCET 383, 1824–1830. <https://doi.org/10.1016/S0140-6736(13)62631-8>
**[17]** Ball, J., Bruyneel, L., Aiken, L., Sermeus, W., Sloane, D., Rafferty, A., Lindqvist, R., Tishelman, C., Griffiths, P., RN4Cast Consortium, 2018. Post-operative mortality, missed care and nurse staffing in nine countries: A cross-sectional study. INTERNATIONAL JOURNAL OF NURSING STUDIES 78, 10–15. <https://doi.org/10.1016/j.ijnurstu.2017.08.004>
**[18]** Salvador, R.O., Gnanlet, A., McDermott, C., 2021. The impact of the use of employee functional flexibility on patient safety. Personnel Review 50, 971–984. <https://doi.org/10.1108/PR-10-2019-0562>
**[19]** Dietermann, K., Winter, V., Schneider, U., Schreyogg, J., 2021. The impact of nurse staffing levels on nursing-sensitive patient outcomes: a multilevel regression approach. EUROPEAN JOURNAL OF HEALTH ECONOMICS 22, 833–846. <https://doi.org/10.1007/s10198-021-01292-2>
**[20]** Needleman, J., Buerhaus, P., Pankratz, V., Leibson, C., Stevens, S., Harris, M., 2011. Nurse Staffing and Inpatient Hospital Mortality. NEW ENGLAND JOURNAL OF MEDICINE 364, 1037–1045. <https://doi.org/10.1056/NEJMsa1001025>
**[21]** Aiken, L., Cimiotti, J., Sloane, D., Smith, H., Flynn, L., Neff, D., 2011. Effects of Nurse Staffing and Nurse Education on Patient Deaths in Hospitals With Different Nurse Work Environments. MEDICAL CARE 49, 1047–1053. <https://doi.org/10.1097/MLR.0b013e3182330b6e>
**[22]** Bettencourt, A., McHugh, M., Sloane, D., Aiken, L., 2020. Nurse Staffing, the Clinical Work Environment, and Burn Patient Mortality. JOURNAL OF BURN CARE & RESEARCH 41, 796–802. <https://doi.org/10.1093/jbcr/iraa061>
**[23]** Ma, C., McHugh, M., Aiken, L., 2015. Organization of Hospital Nursing and 30-Day Readmissions in Medicare Patients Undergoing Surgery. MEDICAL CARE 53, 65–70. <https://doi.org/10.1097/MLR.0000000000000258>
**[24]** Schlak, A., Aiken, L., Chittams, J., Poghosyan, L., McHugh, M., 2021. Leveraging the Work Environment to Minimize the Negative Impact of Nurse Burnout on Patient Outcomes. INTERNATIONAL JOURNAL OF ENVIRONMENTAL RESEARCH AND PUBLIC HEALTH 18. <https://doi.org/10.3390/ijerph18020610>
**[25]** Twigg, D., Duffield, C., Bremner, A., Rapley, P., Finn, J., 2012. Impact of skill mix variations on patient outcomes following implementation of nursing hours per patient day staffing: A retrospective study. Journal of Advanced Nursing 68, 2710–2718. <https://doi.org/10.1111/j.1365-2648.2012.05971.x>
**[26]** Dubois, C.-A., d’Amour, D., Tchouaket, E., Clarke, S., Rivard, M., Blais, R., 2013. Associations of patient safety outcomes with models of nursing care organization at unit level in hospitals. International Journal for Quality in Health Care 25, 110–117. <https://doi.org/10.1093/intqhc/mzt019>
**[27]** Wieczorek-Wojcik, B., Gaworska-Krzeminska, A., Owczarek, A.J., Kilanska, D., 2020. In-hospital mortality as the side effect of missed care. Special Issue: Missed or Rationed Nursing Care 28, 2240–2246. <https://doi.org/10.1111/jonm.12965>
**[28]** Xue Y., Aiken L.H., Freund D.A., Noyes K., 2012. Quality outcomes of hospital supplemental nurse staffing. J. Nurs. Adm. 42, 580–585. <https://doi.org/10.1097/NNA.0b013e318274b5bc>
**[29]** Park S.H., Blegen M.A., Spetz J., Chapman S.A., De Groot H., 2012. Patient turnover and the relationship between nurse staffing and patient outcomes. Res. Nurs. Health 35, 277–288. <https://doi.org/10.1002/nur.21474>
**[30]** Aiken L.H., Shang J., Xue Y., Sloane D.M., 2013. Hospital use of agency-employed supplemental nurses and patient mortality and failure to rescue. Health Serv. Res. 48, 931–948. <https://doi.org/10.1111/1475-6773.12018>
**[31]** Park S.H., Boyle D.K., Bergquist-Beringer S., Staggs V.S., Dunton N.E., 2014. Concurrent and lagged effects of registered nurse turnover and staffing on unit-acquired pressure ulcers. Health Serv. Res. 49, 1205–1225. <https://doi.org/10.1111/1475-6773.12158>
**[32]** Park S.H., Blegen M.A., Spetz J., Chapman S.A., De Groot H.A., 2015. Comparison of nurse staffing measurements in staffing-outcomes research. Med. Care 53, e1–e8. <https://doi.org/10.1097/MLR.0b013e318277eb50>
**[33]** Schreuders L.W., Bremner A.P., Geelhoed E., Finn J., 2015. The relationship between nurse staffing and inpatient complications. J Adv Nurs 71, 800–812. <https://doi.org/10.1111/jan.12572>
**[34]** Giuliano K.K., Danesh V., Funk M., 2016. The Relationship Between Nurse Staffing and 30-Day Readmission for Adults With Heart Failure. J Nurs Adm 46, 25–29. <https://doi.org/10.1097/NNA.0000000000000289>
**[35]** Li X., Bowman S.M., Smith T.C., 2016. Effects of registered nurse staffing level on hospital-acquired conditions in cardiac surgery patients: A propensity score matching analysis. Nurs Outlook 64, 533–541. <https://doi.org/10.1016/j.outlook.2016.05.002>
**[36]** Twigg D.E., Myers H., Duffield C., Pugh J.D., Gelder L., Roche M., 2016. The impact of adding assistants in nursing to acute care hospital ward nurse staffing on adverse patient outcomes: An analysis of administrative health data. Int J Nurs Stud 63, 189–200. <https://doi.org/10.1016/j.ijnurstu.2016.09.008>
**[37]** Haegdorens F., Van Bogaert P., De Meester K., Monsieurs K.G., 2019. The impact of nurse staffing levels and nurse’s education on patient mortality in medical and surgical wards: an observational multicentre study. BMC Health Serv Res 19, 864. <https://doi.org/10.1186/s12913-019-4688-7>
**[38]** Rochefort, C.M., Beauchamp, M.-E., Audet, L.-A., Abrahamowicz, M., Bourgault, P., 2020. Associations of 4 Nurse Staffing Practices With Hospital Mortality. Medical Care 58, 912–918. <https://doi.org/10.1097/MLR.0000000000001397>
**[39]** Fogg C., Bridges J., Meredith P., Spice C., Field L., Culliford D., Griffiths P., 2021. The association between ward staffing levels, mortality and hospital readmission in older hospitalised adults, according to presence of cognitive impairment: A retrospective cohort study. Age Ageing 50, 431–439. <https://doi.org/10.1093/ageing/afaa133>
**[40]** Falk A.-C., 2022. Nurse staffing levels in critical care: The impact of patient characteristics. Nurs Crit Care. <https://doi.org/10.1111/nicc.12826>
**[41]** Roberts K., Thom O., Hocking J., Bernard A., Doyle T., 2022. Clinical incidents in the emergency department: is there an association with emergency nursing shift patterns? A retrospective observational study. BMJ Open Qual. 11, 001785. <https://doi.org/10.1136/bmjoq-2021-001785>
**[42]** Frith KH, Anderson EF, Caspers B, Tseng F, Sanford K, Hoyt NG, Moore K, 2010. Effects of nurse staffing on hospital-acquired conditions and length of stay in community hospitals. Quality Management in Health Care 19, 147–155. <https://doi.org/10.1097/QMH.0b013e3181dafe3f>
**[43]** Trinkof AM, Johantgen M, Storr CL, Gurses AP, Liang Y, Han K, 2011. Nurses’ work schedule characteristics, nurse staffing, and patient mortality. Nursing Research 60, 1–8. <https://doi.org/10.1097/NNR.0b013e3181fff15d>
**[44]** Hart, P., Davis, N., 2011. Effects of nursing care and staff skill mix on patient outcomes within acute care nursing units. Journal of Nursing Care Quality 26, 161–168. <https://doi.org/10.1097/NCQ.0b013e3181efc9cb>
**[45]** Kendall-Gallagher, D., Aiken, L.H., Sloane, D.M., Cimiotti, J.P., 2011. Nurse Specialty Certification, Inpatient Mortality, and Failure to Rescue. Journal of Nursing Scholarship 43, 188–194. <https://doi.org/10.1111/j.1547-5069.2011.01391.x>
**[46]** Staggs, V.S., Knight, J.E., Dunton, N., 2012. Understanding unassisted falls: effects of nurse staffing level and nursing staff characteristics. Journal of Nursing Care Quality 27, 194–199. <https://doi.org/10.1097/ncq.0b013e318241da2d>
**[47]** Boltz, M., Capezuti, E., Wagner, L., Rosenberg, M.-C., Secic, M., 2013. Patient Safety in Medical-Surgical Units: Can Nurse Certification Make a Difference? MEDSURG Nursing 22, 26–37.
**[48]** JiSun Choi, Staggs, V.S., 2014. Comparability of nurse staffing measures in examining the relationship between RN staffing and unit-acquired pressure ulcers: A unit-level descriptive, correlational study. International Journal of Nursing Studies 51, 1344–1352. <https://doi.org/10.1016/j.ijnurstu.2014.02.011>
**[49]** Cottney, A., Innes, J., 2015. Medication-administration errors in an urban mental health hospital: A direct observation study. International Journal of Mental Health Nursing 24, 65–74. <https://doi.org/10.1111/inm.12096>
**[50]** Staggs, V.S., 2016. Deviations in Monthly Staffing and Injurious Assaults Against Staff and Patients on Psychiatric Units. Research in Nursing & Health 39, 347–352. <https://doi.org/10.1002/nur.21735>
**[51]** Rao, A.D., Kumar, A., McHugh, M., 2017. Better Nurse Autonomy Decreases the Odds of 30-Day Mortality and Failure to Rescue. Journal of Nursing Scholarship 49, 73–79. <https://doi.org/10.1111/jnu.12267>
**[52]** Olds, D.M., Aiken, L.H., Cimiotti, J.P., Lake, E.T., 2017. Association of nurse work environment and safety climate on patient mortality: A cross-sectional study. International Journal of Nursing Studies 74, 155–161. <https://doi.org/10.1016/j.ijnurstu.2017.06.004>
**[53]** Harrison, J.M., Aiken, L.H., Sloane, D.M., Brooks Carthon, J.M., Merchant, R.M., Berg, R.A., McHugh, M.D., 2019. In Hospitals With More Nurses Who Have Baccalaureate Degrees, Better Outcomes For Patients After Cardiac Arrest. Health Affairs 38, 1087–1094. <https://doi.org/10.1377/hlthaff.2018.05064>
**[54]** Needleman, J., Liu, J., Shang, J., Larson, E.L., Stone, P.W., 2020. Association of registered nurse and nursing support staffing with inpatient hospital mortality. BMJ Qual Saf 29, 10–18. <https://doi.org/10.1136/bmjqs-2018-009219>
**[55]** Dall’Ora, C., Maruotti, A., Griffiths, P., 2020. Temporary Staffing and Patient Death in Acute Care Hospitals: A Retrospective Longitudinal Study. Journal of Nursing Scholarship 52, 210–216. <https://doi.org/10.1111/jnu.12537>
**[56]** Flanagan, J.M., Read, C., Shindul-Rothschild, J., 2020. Factors Associated With the Rate of Sepsis After Surgery. Critical Care Nurse 40, e1–e9. <https://doi.org/10.4037/ccn2020171>
**[57]** Lake, E.T., Shang, J., Klaus, S., Dunton, N.E., 2010. Patient falls: Association with hospital Magnet status and nursing unit staffing. Res Nurs Health 33, 413–425. <https://doi.org/10.1002/nur.20399>
**[58]** Bae, S.-H., Mark, B., Fried, B., 2010. Use of temporary nurses and nurse and patient safety outcomes in acute care hospital units. Health Care Manage Rev 35, 333–344. <https://doi.org/10.1097/HMR.0b013e3181dac01c>
**[59]** Esparza, S.J., Zoller, J.S., White, A.W., Highfield, M.E.F., 2012. Nurse staffing and skill mix patterns: Are there differences in outcomes? J Healthc Risk Manag 31, 14–23. <https://doi.org/10.1002/jhrm.20092>
**[60]** Wiltse Nicely, K.L., Sloane, D.M., Aiken, L.H., 2013. Lower mortality for abdominal aortic aneurysm repair in high-volume hospitals is contingent upon nurse staffing. Health Serv Res 48, 972–991. <https://doi.org/10.1111/1475-6773.12004>
**[61]** Choi, J., Bergquist-Beringer, S., Staggs, V.S., 2013. Linking RN workgroup job satisfaction to pressure ulcers among older adults on acute care hospital units. Res Nurs Health 36, 181–190. <https://doi.org/10.1002/nur.21531>
**[62]** Talsma, A., Jones, K., Guo, Y., Wilson, D., Campbell, D.A., 2014. The relationship between nurse staffing and failure to rescue: where does it matter most? J Patient Saf 10, 133–139. <https://doi.org/10.1097/PTS.0b013e31829954e2>
**[63]** Kelly, D.M., Kutney-Lee, A., McHugh, M.D., Sloane, D.M., Aiken, L.H., 2014. Impact of critical care nursing on 30-day mortality of mechanically ventilated older adults. Crit Care Med 42, 1089–1095. <https://doi.org/10.1097/CCM.0000000000000127>
**[64]** Ma, C., Park, S.H., 2015. Hospital Magnet Status, Unit Work Environment, and Pressure Ulcers. J Nurs Scholarsh 47, 565–573. <https://doi.org/10.1111/jnu.12173>
**[65]** McHugh, M.D., Rochman, M.F., Sloane, D.M., Berg, R.A., Mancini, M.E., Nadkarni, V.M., Merchant, R.M., Aiken, L.H., 2016. Better Nurse Staffing and Nurse Work Environments Associated With Increased Survival of In-Hospital Cardiac Arrest Patients. Med Care 54, 74–80. <https://doi.org/10.1097/MLR.0000000000000456>
**[66]** Drews, F.A., Markewitz, B.A., Stoddard, G.J., Samore, M.H., 2019. Interruptions and Delivery of Care in the Intensive Care Unit. Hum Factors 61, 564–576. <https://doi.org/10.1177/0018720819838090>
**[67]** Shang, J., Needleman, J., Liu, J., Larson, E., Stone, P.W., 2019. Nurse Staffing and Healthcare-Associated Infection, Unit-Level Analysis. J Nurs Adm 49, 260–265. <https://doi.org/10.1097/NNA.0000000000000748>
**[68]** Margadant, C., Wortel, S., Hoogendoorn, M., Bosman, R., Spijkstra, J.J., Brinkman, S., de Keizer, N., 2020. The Nursing Activities Score Per Nurse Ratio Is Associated With In-Hospital Mortality, Whereas the Patients Per Nurse Ratio Is Not. Crit Care Med 48, 3–9. <https://doi.org/10.1097/CCM.0000000000004005>
**[69]** Lasater, K.B., McHugh, M., Rosenbaum, P.R., Aiken, L.H., Smith, H., Reiter, J.G., Niknam, B.A., Hill, A.S., Hochman, L.L., Jain, S., Silber, J.H., 2021. Valuing hospital investments in nursing: multistate matched-cohort study of surgical patients. BMJ Qual Saf 30, 46–55. <https://doi.org/10.1136/bmjqs-2019-010534>
**[70]** Juvé-Udina, M.-E., González-Samartino, M., López-Jiménez, M.M., Planas-Canals, M., Rodríguez-Fernández, H., Batuecas Duelt, I.J., Tapia-Pérez, M., Pons Prats, M., Jiménez-Martínez, E., Barberà Llorca, M.À., Asensio-Flores, S., Berbis-Morelló, C., Zuriguel-Pérez, E., Delgado-Hito, P., Rey Luque, Ó., Zabalegui, A., Fabrellas, N., Adamuz, J., 2020. Acuity, nurse staffing and workforce, missed care and patient outcomes: A cluster-unit-level descriptive comparison. J Nurs Manag 28, 2216–2229. <https://doi.org/10.1111/jonm.13040>
**[71]** Lasater, K.B., McHugh, M.D., Rosenbaum, P.R., Aiken, L.H., Smith, H.L., Reiter, J.G., Niknam, B.A., Hill, A.S., Hochman, L.L., Jain, S., Silber, J.H., 2021. Evaluating the Costs and Outcomes of Hospital Nursing Resources: a Matched Cohort Study of Patients with Common Medical Conditions. J Gen Intern Med 36, 84–91. <https://doi.org/10.1007/s11606-020-06151-z>
**[72]** Porcel-Gálvez, A.M., Barrientos-Trigo, S., Gil-García, E., Aguilera-Castillo, O., Pérez-Fernández, A.J., Fernández-García, E., 2020. Factors Associated with In-Hospital Mortality in Acute Care Hospital Settings: A Prospective Observational Study. Int J Environ Res Public Health 17. <https://doi.org/10.3390/ijerph17217951>
**[73]** Dzikowicz, D.J., Schmitt, L.A., Gastle, K., Skermont, A., Carey, M.G., 2020. Comparing an All-RN Unit to a Mixed-Skill Unit at a Hospital. J Nurs Adm 50, e14–e22. <https://doi.org/10.1097/NNA.0000000000000954>
**[74]** Lasater, K.B., Sloane, D.M., McHugh, M.D., Cimiotti, J.P., Riman, K.A., Martin, B., Alexander, M., Aiken, L.H., 2021. Evaluation of hospital nurse-to-patient staffing ratios and sepsis bundles on patient outcomes. Am J Infect Control 49, 868–873. <https://doi.org/10.1016/j.ajic.2020.12.002>
**[75]** Fasoi, G., Patsiou, E.C., Stavropoulou, A., Kaba, E., Papageorgiou, D., Toylia, G., Goula, A., Kelesi, M., 2020. Assessment of Nursing Workload as a Mortality Predictor in Intensive Care Units (ICU) Using the Nursing Activities Score (NAS) Scale. Int J Environ Res Public Health 18. <https://doi.org/10.3390/ijerph18010079>
**[76]** Scheier, T., Kuster, S.P., Dunic, M., Falk, C., Sax, H., Schreiber, P.W., 2021. Does continuity in nursing staff matter? A pilot study on correlation of central line-associated bloodstream infections and employee turnover. Antimicrob Resist Infect Control 10, 90. <https://doi.org/10.1186/s13756-021-00958-z>

**[77]** Kumar, A., Sloane, D., Aiken, L., McHugh, M., 2022. Hospital nursing factors associated with decreased odds of mortality in older adult medicare surgical patients with depression. BMC Geriatr 22, 665. <https://doi.org/10.1186/s12877-022-03348-1>

**[78]** Moronski, L.S., Lake, E.T. and McHugh, M.D. (2025), Associations Between Nurse Staffing Levels and 30- and 60-Day Readmissions for Acute Care Patients With Intellectual and Developmental Disability. Research in Nursing & Health, 48: 675-684. <https://doi.org/10.1002/nur.70013>

**[79]** Fernández-Sánchez, Juan David, Andrés-Pretel, Fernando, Ortiz-Diaz, Verónica, Molina-Alarcón, Milagros, Lozano-Hernández, Cristina M., Relationship Between the Nurse–Patient Ratio and Adverse Events in Conventional Hospitalization Units in a Third-Level Hospital, Journal of Nursing Management, 2025, 8885593, 15 pages, 2025. <https://doi.org/10.1155/jonm/8885593>

**[80]** Lasater KB, Brom H, Aiken LH*, et al*., Are minimum nurse-to-patient staffing ratios needed in hospitals? An observational study in British Columbia, Canada, *BMJ Open* 2025;**15:**e099358. doi: <https://10.1136/bmjopen-2025-099358>

**[81]** Iroegbu, C., Kutney-Lee, A., Chittams, J., Leak, S. and Brooks-Carthon, M. (2026), The Impact of Nurse Staffing and Education on 30-Day Mortality Among Patients Hospitalized for Acute Kidney Injury. Research in Nursing & Health, 49: 19-27. <https://doi.org/10.1002/nur.70024>

**[82]** Tack, J., Bruyneel, A., Maes, J., Mercier, G., Taccone, F.S. and Pirson, M. (2026), Association Between Nursing Workload and Intensive Care Unit Readmissions: A Prospective Cohort Study. J Adv Nurs, 82: 1300-1312. <https://doi.org/10.1111/jan.16984>

**[83]** Moscelli G, Mello M, Sayli M, Boyle A. Nurse and doctor turnover and patient outcomes in NHS acute trusts in England: retrospective longitudinal study *BMJ* 2024; 387 :e079987 <https://doi:10.1136/bmj-2024-079987>

**[84]** K. Jane Muir, Daniela Golinelli, Karen B. Lasater, Linda H. Aiken, Raina Merchant, Matthew D. McHugh, J. Margo Brooks Carthon,

Association of Emergency Department and Medical-Surgical Nurse Staffing on Patient Departures Against Medical Advice,

Journal of Emergency Nursing, 2026, ISSN 0099-1767, <https://doi.org/10.1016/j.jen.2025.12.009>
